# Deletion of *Tmem268* in mice suppresses anti-infectious immune responses by downregulating CD11b signaling

**DOI:** 10.1038/s44319-024-00141-6

**Published:** 2024-05-10

**Authors:** Mengyuan Duan, Xuan Zhang, Yaxin Lou, Jinqiu Feng, Pengli Guo, Shufang Ye, Ping Lv, Yingyu Chen

**Affiliations:** 1 Department of Immunology, Peking University School of Basic Medical Sciences; NHC Key Laboratory of Medical Immunology, Peking University, 38 Xueyuan Road, 100191 Beijing, China; 2 Beijing Key Laboratory for Pediatric Diseases of Otolaryngology, Beijing Pediatric Research Institute, Capital Medical University, National Center for Children’s Health, 100045 Beijing, China; 3 Medical and Healthy Analytical Center, Peking University, 38 Xueyuan Road, 100191 Beijing, China; 4 Center for Human Disease Genomics, Peking University, 38 Xueyuan Road, 100191 Beijing, China

**Keywords:** TMEM268, CD11b, Phagocytes, Integrin, Sepsis, Immunology, Molecular Biology of Disease

## Abstract

Transmembrane protein 268 (TMEM268) is a novel, tumor growth-related protein first reported by our laboratory. It interacts with the integrin subunit β4 (ITGB4) and plays a positive role in the regulation of the ITGB4/PLEC signaling pathway. Here, we investigated the effects and mechanism of TMEM268 in anti-infectious immune response in mice. *Tmem268* knockout in mice aggravated cecal ligation and puncture-induced sepsis, as evidenced by higher bacterial burden in various tissues and organs, congestion, and apoptosis. Moreover, *Tmem268* deficiency in mice inhibited phagocyte adhesion and migration, thus decreasing phagocyte infiltration at the site of infection and complement-dependent phagocytosis. Further findings indicated that TMEM268 interacts with CD11b and inhibits its degradation via the endosome–lysosome pathway. Our results reveal a positive regulatory role of TMEM268 in β2 integrin-associated anti-infectious immune responses and signify the potential value of targeting the TMEM268–CD11b signaling axis for the maintenance of immune homeostasis and immunotherapy for sepsis and related immune disorders.

## Synopsis

TMEM268 interacts with the β2 integrin subunit CD11b and attenuates its degradation via the endosome–lysosome pathway, thereby promoting phagocyte adhesion, migration, phagocytosis and bacterial clearance during sepsis.

- *Tmem268*-deficiency impairs bacterial clearance and aggravates CLP-induced sepsis.
- *Tmem268*-deficiency suppresses phagocyte adhesion, migration and phagocytosis by downregulating CD11b.
- *Tmem268* knockout promotes CD11b degradation via the endosomal–lysosomal pathway.

## Abstract (web summary)

TMEM268 interacts with the β2 integrin subunit CD11b and attenuates its degradation via the endosome–lysosome pathway, thereby promoting phagocyte adhesion, migration, phagocytosis and bacterial clearance during sepsis.

## Graphical abstract

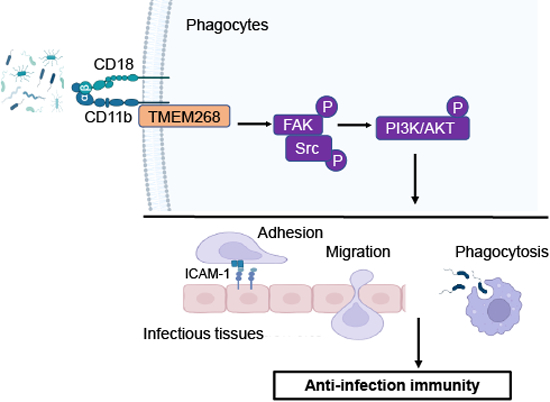

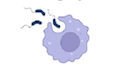

## Introduction

Innate immunity acts as the first line of defense against invading pathogens by sensing and responding to pathogen-associated molecular patterns (PAMPs) and endogenous damage-associated molecular patterns (DAMPs). The pattern recognition receptors (PRRs) in innate immune cells are a key element of the immune system, including Toll-like receptors (TLRs), Nod-like receptors (NLRs), C-type lectin receptors, and intracellular DNA and RNA sensors. Upon the recognition of their specific ligands from invasive pathogens (e.g., bacteria, viruses, and fungi) or damaged cells, PRRs initiate various downstream signaling cascades, including nuclear factor kappa B (NF-kB), type I interferon (IFN), and inflammasome signaling pathways, leading to the production of corresponding proinflammatory cytokines or chemokines (Brubaker et al, 2015; Fitzgerald and Kagan, 2020). The activation of TLR signaling is also crucial to the induction of antigen-specific adaptive immune responses by activating antigen-presenting cells (e.g., macrophages and dendritic cells) and inducing adaptive immune cells for the clearance of invading pathogens. Persistent infection triggers excessive inflammation and cellular injury, which may result in decreased bacterial clearance and lead to organ dysfunction and failure (Singer et al, 2016; van der Poll et al, 2017).

CD11b/CD18, also named MAC-1 or complement receptor 3 (CR3), is a heterodimer belonging to the β2 integrin family and composed of the integrin α subunit CD11b and β subunit CD18 (Schittenhelm et al, 2017). It is an important adhesion molecule on immune cell membranes, which mediates various biological activities, including adhesion, migration, phagocytosis, and apoptosis of immune cells. It plays a crucial role in the host immune responses against infection (Arnaout, 2016; Bednarczyk et al, 2020; Yuki and Hou, 2020). Abnormal expression or dysfunction of CD11b/CD18 are closely associated with numerous autoimmune and infectious diseases; thus, their protein homeostasis must be finely regulated, which facilitates the recruitment of and phagocytosis by phagocytes at the sites of infection. Several studies have identified positive regulators of β2 integrins, which regulate the expression or activation of β2 integrins through different mechanisms. For instance, the immunomodulatory lectin galectin-9 (Gal-9) released by activated vascular endothelial cells was demonstrated to increase the β2 integrin expression on neutrophils by binding with CD44, which in turn strengthens the neutrophil–endothelial interaction and promotes the recruitment of neutrophils during inflammation, suggesting the pro-adhesive effects of Gal-9 (Iqbal et al, 2022). Another proinflammatory mediator, myeloid-related protein 8 and 14 (Mrp8/14), was identified as a key modulator of the leukocyte recruitment cascade. Mrp8/14 activates a TLR4-mediated, Rap1-GTPase-dependent pathway of rapid β2 integrin activation in neutrophils, thus promoting the adhesion of neutrophils to vascular endothelium in case of inflammation (Pruenster et al, 2015). To date, the regulatory mechanisms for the expression of MAC-1 protein remain largely unknown.

Transmembrane protein 268 (TMEM268) is a novel tumor growth-related protein first identified by our laboratory (Hong et al, 2019). Our previous studies have demonstrated that TMEM268 promotes the adhesion ability of tumor cells by positively regulating the β4 integrin signaling pathway, thereby promoting the occurrence and development of gastric cancer. However, the role of TMEM268 in innate immune responses remains to be investigated. Bioinformatics analysis suggests that human *TMEM268* is highly expressed in monocytes and macrophages (https://www.proteinatlas.org/ENSG00000157693-TMEM268). In addition, the TLR4 ligand lipopolysaccharide (LPS) has been shown to significantly decrease the expression of *Tmem268* in peritoneal macrophages (http://biogps.org/#goto=genereport&id=230279), indicating that TMEM268 may be involved in the regulation of inflammatory immune responses.

In this study, we demonstrated that *Tmem268* deficiency in mice inhibits the adhesion and migration of phagocytes, decreasing the recruitment of phagocytes at the site of infection, thereby impairing bacterial clearance through phagocytosis. Furthermore, the findings suggest that TMEM268 interacts with the C-terminus of CD11b and inhibits its degradation via the endosomal–lysosomal pathway. Moreover, *Tmem268* ablation significantly blocks the FAK/Src signaling pathway downstream of CD11b in mouse macrophages. These results indicate the positive regulatory role of TMEM268 in CD11b/CD18-associated anti-infectious immune responses.

## Results

### *Tmem268* deficiency impairs bacterial clearance and aggravates cecal ligation and puncture-induced sepsis

We first evaluated the expression profile of *Tmem268* in mouse immune cells. Data from quantitative reverse-transcription PCR (qRT-PCR) indicated that *Tmem268* was abundantly expressed in monocytes and macrophages, and its expression was significantly lower in LPS-stimulated cells (Appendix Fig. S1). These results suggest a potential function for TMEM268 in the inflammatory response.

To further investigate the biological activities of TMEM268, we generated *Tmem268*-deficient (*Tmem268*^*−/−*^) mice using CRISPR–Cas9 technology (Appendix Fig. S2). Flow cytometry data indicated that the proportion of B cells, T cells, monocytes, neutrophils, and macrophages in different tissues were comparable between *Tmem268*^*+/+*^ and *Tmem268*^*−/−*^ mice, suggesting that *Tmem268* knockout did not affect immunocyte development and homeostasis (Appendix Fig. S3). Next, we established an endotoxic shock model induced by intraperitoneal injection with LPS in mice. We found that *Tmem268* ablation induced the LPS-induced inflammation response, as evidenced by the shorter survival time, severe pulmonary hemorrhage in lung tissues, and higher serum level of TNF-ɑ in *Tmem268*^*−/−*^ mice (Fig. EV1A–D).

**Figure EV1:**

*Tmem268* ablation exacerbates LPS- and CLP-induced inflammation, increases the bacterial burdens in reconstituted chimeric mice. (**A**) Survival curve of *Tmem268*^*+/+*^ and *Tmem268*^*−/−*^ mice (*n* = 5) intraperitoneally injected with LPS (13 mg/kg). **P value = 0.0017, the Log-rank (Mantel–Cox) test. (**B**, **C**) Serum levels of TNF-α and IL-6 in mice intraperitoneally injected with LPS (5 mg/kg) for indicated time. Mean ± SD (*n* = 3). Unpaired two-tailed *t* test. For LPS 1 h, *P value = 0.0433. (**D**) H&E staining of *Tmem268*^*+/+*^ and *Tmem268*^*−/−*^ lung after LPS injection for 24 h. Scale bars = 50 μm. (**E**) Serum levels of TNF-α, IFN-β, MCP-1/CCL2 in mice after CLP surgery for 8 h. Mean ± SD (*n* = 3). Unpaired two-tailed *t* test. Mean ± SD. For TNF-α, ***P value = 0.0008. For IFN-β, ***P value = 0.0006. For MCP-1/CCL2, *P value = 0.0157. (**F**) The bacterial burdens were determined in different chimeric mice with CLP at 24 h. (**G**) H&E staining of lung in reconstituted chimeric mice with CLP at 24 h. Scale bars = 50 μm.

**Figure 1:**
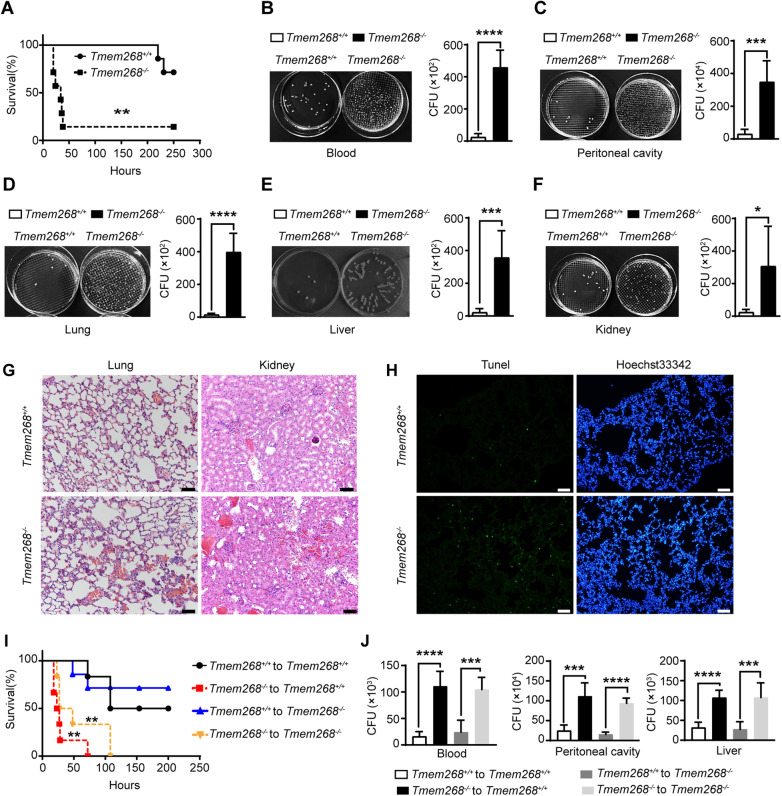
*Tmem268* deficiency aggravates CLP-induced sepsis. (**A**) Survival curve of *Tmem268*^*+/+*^ and *Tmem268*^*−/−*^ mice subjected to CLP (*n* = 7). **P value = 0.0059, the Log-rank (Mantel–Cox) test. (**B**–**F**) The bacterial burdens were determined in different mice with CLP at 24 h. (**B**) blood, ****P < 0.0001, (**C**) peritoneal cavity, ***P value = 0.0001, (**D**) lung, ****P < 0.0001, (**E**) liver, ***P value = 0.0006, (**F**) kidney, *P value = 0.0176. Unpaired two-tailed *t* test. Mean ± SD (*n* = 6 mice). (**G**) H&E staining of lung and kidney in *Tmem268*^*+/+*^ and *Tmem268*^*−/−*^ mice with CLP at 24 h. Scale bars = 50 μm. (**H**) TUNEL staining of lung in *Tmem268*^*+/+*^ and *Tmem268*^*−/−*^ mice with CLP at 24 h. Scale bars = 50 μm. (**I**) Survival curve of *Tmem268*^*+/+*^ (WT) and *Tmem268*^*−/−*^ (KO) chimeras mice reconstituted with *Tmem268*^*+/+*^ or *Tmem268*^*−/−*^ bone marrow subjected to CLP (*n* = 6). WT → WT vs KO → WT, **P value = 0.0011; WT → KO vs KO → KO, **P value = 0.0092. The Log-rank (Mantel–Cox) test. (**J**) The bacterial burdens were determined in different chimeric mice with CLP at 24 h. For blood, ****P < 0.0001, ***P value = 0.0001. For peritoneal cavity, ***P value = 0.0002, ****P < 0.0001. For liver, ****P < 0.0001, ***P value = 0.0009. Unpaired two-tailed *t* test. Mean ± SD (*n* = 6 mice). Source data are available online for this figure.

Next, we subjected *Tmem268*^*+/+*^ and *Tmem268*^*−/−*^ mice to cecal ligation and puncture (CLP), to establish a clinically relevant sepsis model of polymicrobial infection (Stearns-Kurosawa et al, 2011). We found that the *Tmem268*^*−/−*^ mice showed a significantly higher mortality than their *Tmem268*^*+/+*^ littermates; at 24 h after CLP, 43% of all *Tmem268*^*−/−*^ mice died, whereas all *Tmem268*^*+/+*^ mice were alive. At 231 h after CLP, only 14% of *Tmem268*^*−/−*^ mice were alive, whereas 71% of *Tmem268*^*+/+*^ mice continued to survive (Fig. 1A). The bacterial burden in various tissues and organs was examined at 24 h post CLP. Compared to *Tmem268*^*+/+*^ mice, *Tmem268*^*−/−*^ mice showed significantly higher bacterial burden in lung, liver, kidney, blood, and peritoneal cavity (Fig. 1B–F). Moreover, *Tmem268*^*−/−*^ mice showed significantly higher serum levels of the proinflammatory cytokines tumor necrosis factor (TNF)-α, IFN-β, and chemokine MCP-1/CCL2 (Fig. EV1E). Consistent with these observations, *Tmem268*^*−/−*^ mice displayed severely damaged lung and kidney tissues (Fig. 1G) and a higher number of apoptotic cells in lung tissue (Fig. 1H).

Because TMEM268 is expressed by both stromal and myeloid cells, we created bone marrow chimeras to investigate whether stromal or hematopoietic TMEM268 is required in antibacterial defense. To be specific, *Tmem268*^*+/+*^ or *Tmem268*^*−/−*^ bone marrow (BM) was transplanted into lethally irradiated *Tmem268*^*+/+*^ or *Tmem268*^*−/−*^ mice, and their response to CLP was evaluated. We found that among the 4 groups of chimeras, mice received from *Tmem268*^*−/−*^ BM (*Tmem268*^*−/−*^ to *Tmem268*^*+/+*^, *Tmem268*^*−/−*^ to *Tmem268*^*−/−*^) exhibited a worse sepsis phenotype than mice that received *Tmem268*^*+/+*^ BM (*Tmem268*^*+/+*^ to *Tmem268*^*+/+*^, *Tmem268*^*+/+*^ to *Tmem268*^*−/−*^), as evidenced by the higher mortality (Fig. 1I), higher bacterial load (Figs. EV1F and  1J) and more severe lung injury (Fig. EV1G). There was no significant difference between *Tmem268*^*+/+*^ and *Tmem268*^*−/−*^ recipients which received from the *Tmem268*^*+/+*^ donor bone marrow, indicating that the *Tmem268*^*+/+*^ immune cells to *Tmem268*^*−/−*^ mice significantly alleviated the phenotype of sepsis (Figs. 1I,J and EV1F,G). These results suggest that hematopoietic TMEM268 positively regulates the antibacterial immune response. Taken together, these findings suggest that *Tmem268* deficiency impairs bacterial clearance and aggravates CLP-induced organ failure.

Since *Tmem268* expression is silenced in all cells of *Tmem268*^*−/−*^ mice, we wanted to investigate whether macrophages and neutrophils are involved in *Tmem268*-mediated effects. *Tmem268*^+/+^or *Tmem268*^−/−^ mice were intraperitoneally injected with clodronate liposome, and F4/80^+^ macrophages in the peritoneal cavity were analyzed by flow cytometry at 72 h post treatment. As shown in Fig. EV2A, clodronate liposome administration significantly decreased the number of F4/80^+^ macrophages, indicating effective clearance of mouse macrophages. Subsequently, mice were subjected to CLP for 8 h. As illustrated in Fig. EV2C, depletion of macrophages in *Tmem268*^*−/−*^ CLP mice still had higher bacterial load than that in *Tmem268*^*+/+*^ CLP mice. In addition, the depletion of neutrophils in *Tmem268*^*−/−*^ CLP mice also displayed a higher bacterial load than that in *Tmem268*^*+/+*^ CLP mice (Fig. EV2B,D). Interestingly, similar results were also observed in *Tmem268*^*−/−*^ CLP mice that underwent the depletion of both macrophages and neutrophils (Fig. EV2E). These results suggest that in addition to macrophages and neutrophils, another mechanism may also be responsible for the bacterial clearance in CLP-treated *Tmem268* KO mice.

**Figure EV2:**

Both macrophages and neutrophils are involved in TMEM268-mediated antibacterial effects. (**A**) The percentage of CD11b^+^F4/80^+^ macrophages in peritoneal cavity were analyzed by flow cytometry from *Tmem268*^*+/+*^ and *Tmem268*^*−/−*^ mice intraperitoneally injected with PBS or clodronate liposome at 72 h. (**B**) The percentage of CD11b^+^Ly6G^+^ neutrophils in bone marrow were analyzed by flow cytometry from *Tmem268*^*+/+*^ and *Tmem268*^*−/−*^ mice intraperitoneally injected with IgG isotype antibody or anti-Ly6G antibody at 48 h. (**C**) *Tmem268*^*+/+*^ and *Tmem268*^*−/−*^ mice pre-treated with PBS or clodronate liposome were subjected to CLP. 8 h later, the bacterial burdens in liver, lung and peritoneal cavity were measured. Mean ± SD (*n* = 3). Unpaired two-tailed *t* test. *P < 0.05, **P < 0.01, ***P < 0.001, ****P < 0.0001. (**D**) *Tmem268*^*+/+*^ and *Tmem268*^*−/−*^ mice pre-treated with IgG isotype or anti-Ly6G antibody were subjected to CLP. 8 h later, the bacterial burdens in liver, lung and peritoneal cavity were measured. Mean ± SD (*n* = 3). Unpaired two-tailed *t* test. **P < 0.01, ***P < 0.001, (**E**) *Tmem268*^*+/+*^ and *Tmem268*^*−/−*^ mice pre-treated with PBS+IgG isotype or liposome+anti-Ly6G antibody were subjected to CLP. 8 h later, the bacterial burdens in liver, lung and peritoneal cavity were measured. Mean ± SD (*n* = 3). Unpaired two-tailed *t* test. ***P < 0.001, ****P < 0.0001.

### *Tmem268* knockout reduces phagocyte infiltration in cecal ligation and puncture-induced sepsis

**Figure 2:**
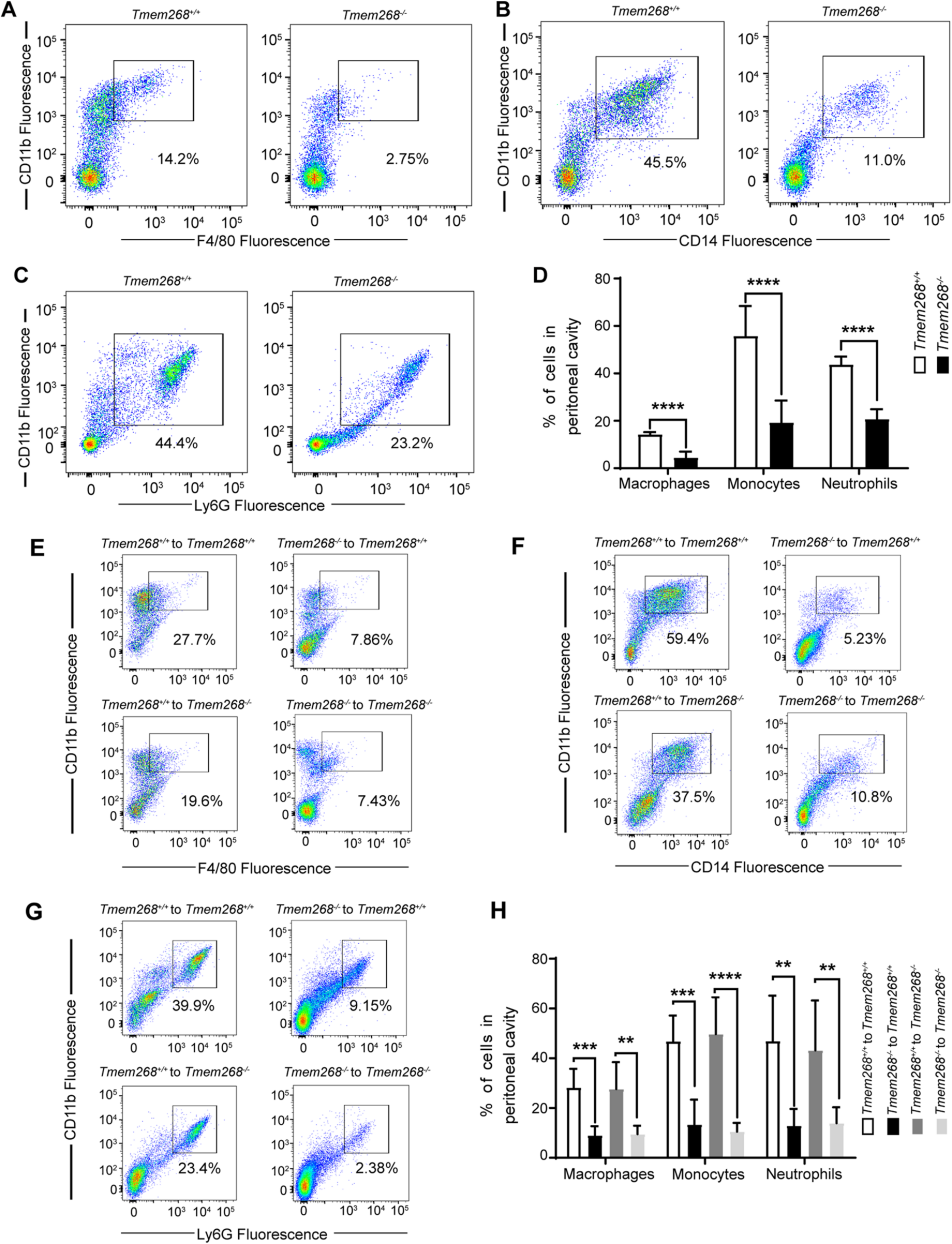
*Tmem268* knockout reduces phagocyte infiltration in CLP-induced sepsis. (**A**–**C**) The percentage of CD11b^+^F4/80^+^ macrophages (**A**), CD11b^+^CD14^+^ monocytes (**B**), and CD11b^+^Ly6G^+^ neutrophils (**C**) were analyzed by flow cytometry in the peritoneal cavity of *Tmem268*^*+/+*^ and *Tmem268*^*−/−*^ mice with CLP at 8 h. (**D**) Quantification of proportions of macrophages, monocytes and neutrophils in *Tmem268*^*+/+*^ and *Tmem268*^*−/−*^ mice. ****P < 0.0001. Unpaired two-tailed *t* test. Mean ± SD (*n* = 7 mice). (**E**–**G**) The percentage of CD11b^+^F4/80^+^ macrophages (**E**), CD11b^+^CD14^+^ monocytes (**F**) and CD11b^+^Ly6G^+^ neutrophils (**G**) were analyzed by flow cytometry in peritoneal cavity of *Tmem268*^*+/+*^ and *Tmem268*^*−/−*^ reconstituted chimeras with CLP at 8 h. (**H**) Quantification of proportions of macrophages, monocytes and neutrophils in *Tmem268*^*+/+*^ and *Tmem268*^*−/−*^ reconstituted chimeras. Mean ± SD (*n* = 6). **P < 0.01, ***P < 0.001, ****P < 0.0001. Unpaired two-tailed *t* test. Source data are available online for this figure.

Upon initiation of infection or inflammation, phagocytes are recruited to the affected site in response to various chemokines, which ultimately contribute to the elimination of invading pathogens (Sun et al, 2021). Therefore, we determined the proportion of phagocytes in the peritoneal cavity of both *Tmem268*^*+/+*^ and *Tmem268*^*−/−*^ CLP mice using flow cytometry. As shown in Fig. 2A–D, the proportion of CD11b^+^F4/80^+^ macrophages, CD11b^+^CD14^+^ monocytes and CD11b^+^Ly6G^+^ neutrophils in the peritoneal cavity was significantly lower in *Tmem268*^*−/−*^ CLP mice than in *Tmem268*^*+/+*^ CLP mice. However, the percentage of monocytes and neutrophils in blood was significantly higher in *Tmem268*^*−/−*^ CLP mice than in *Tmem268*^*+/+*^ CLP mice (Fig. EV3A–C). Furthermore, chimeric mice received from *Tmem268*^*−/−*^ bone marrow (*Tmem268*^*−/−*^ to *Tmem268*^*+/+*^, *Tmem268*^*−/−*^ to *Tmem268*^*−/−*^) also showed less macrophages, monocytes, and neutrophils accumulation in the peritoneal cavity than chimeric mice possessing *Tmem268*^*+/+*^ bone marrow (*Tmem268*^*+/+*^ to *Tmem268*^*+/+*^, *Tmem268*^*+/+*^ to *Tmem268*^*−/−*^) (Fig. 2E–H). Simultaneously, the percentage of monocytes and neutrophils in blood was increased in chimeric mice reconstituted with *Tmem268*^*−/−*^ bone marrow (Fig. EV3D–F). These results suggest that *Tmem268* deficiency inhibits phagocyte recruitment to the site of infection.

**Figure EV3:**

*Tmem268* deficiency increases the proportions of monocytes and neutrophils in peripheral blood. (**A**, **B**) The proportions of CD11b^+^CD14^+^ monocytes (**A**), CD11b^+^Ly6G^+^ neutrophils (**B**) in peripheral blood were detected by flow cytometry in *Tmem268*^*+/+*^ and *Tmem268*^*−/−*^ mice after CLP surgery for 8 h. (**C**) Quantification of monocytes and neutrophils in *Tmem268*^*+/+*^ and *Tmem268*^*−/−*^ mice after CLP surgery for 8 h. For monocytes, **P value = 0.0066. For neutrophils, **P value = 0.0026. Unpaired two-tailed *t* test. Mean ± SD (*n* = 6 mice). (**D**, **E**) The proportions of CD11b^+^CD14^+^ monocytes (**D**), CD11b^+^Ly6G^+^ neutrophils (**E**) in peripheral blood were detected by flow cytometry in *Tmem268*^*+/+*^ and *Tmem268*^*−/−*^ reconstituted chimeras after CLP surgery for 8 h. (**F**) Quantification of monocytes and neutrophils in *Tmem268*^*+/+*^ and *Tmem268*^*−/−*^ reconstituted chimeras after CLP surgery for 8 h. *P < 0.05, **P < 0.01, ***P < 0.001. Unpaired two-tailed *t* test. Mean ± SD (*n* = 6 mice).

### *Tmem268* knockout impairs CD11b/CD18-mediated phagocytosis

**Figure 3:**
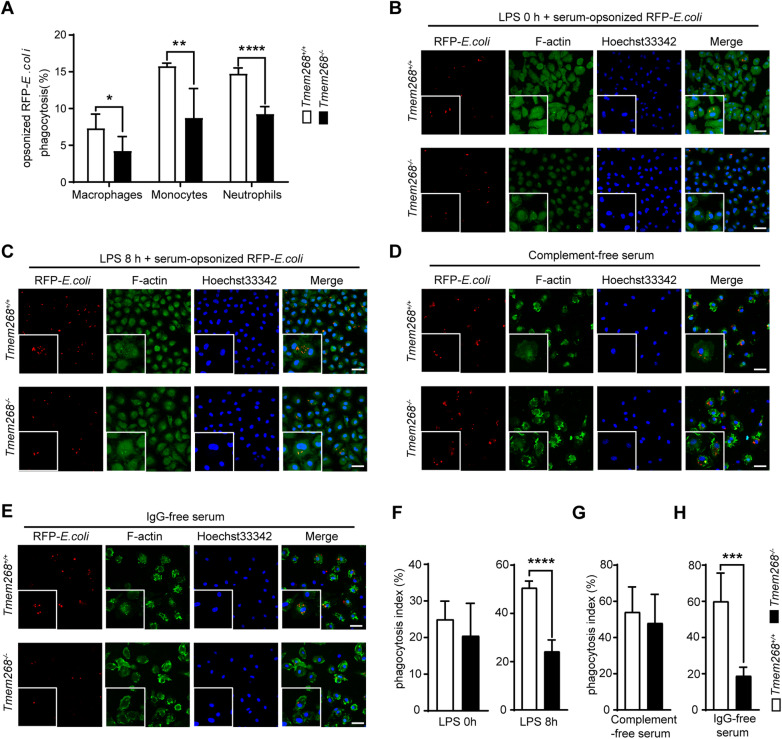
*Tmem268* knockout impairs CR3-mediated phagocytosis. (**A**) *Tmem268*^*+/+*^ and *Tmem268*^*−/−*^ mice pre-treated with LPS for 2 h were intraperitoneally injected with serum-opsonized RFP-*E.coli*. 30 min later, the proportions of RFP^+^F4/80^+^, RFP^+^Ly6C^+^ and RFP^+^Ly6G^+^ cells in the peritoneal cavity were analyzed by flow cytometry. For macrophages, *P value = 0.0356. For monocytes, **P value = 0.0043. For neutrophils, ****P < 0.0001. Unpaired two-tailed *t* test. Mean ± SD (*n* = 5 mice). (**B**, **C**) *Tmem268*^*+/+*^ and *Tmem268*^*−/−*^ BMDMs were treated with or without LPS for 8 h, then cultured with serum-opsonized RFP-*E.coli* for 30 min. The representative fluorescence images were obtained from confocal microscopy. Scale bars = 20 μm. (**D**, **E**) LPS-stimulated *Tmem268*^*+/+*^ and *Tmem268*^*−/−*^ BMDMs were cultured with complement-free (**D**) or IgG-free (**E**) serum-opsonized RFP-*E.coli* for 30 min. The representative fluorescence images were obtained from confocal microscopy. Scale bars = 20 μm. (**F**) The phagocytosis index of BMDMs in (**B**, **C**). For LPS 8 h, ****P < 0.0001, Data (mean ± SD) are representative of at least three independent experiments. Unpaired two-tailed *t* test. (**G**, **H**) The phagocytosis index of BMDMs in (**D**, **E**), respectively. For IgG-free serum, ***P value = 0.0004. Data (mean ± SD) are representative of at least three independent experiments. Unpaired two-tailed *t* test. Source data are available online for this figure.

CD11b/CD18, also named complement receptor 3 (CR3), plays an important role in the removal of invading pathogens via C3-opsonized phagocytosis in phagocytes (Lamers et al, 2021). Therefore, we examined phagocytosis in *Tmem268*^*−/−*^ phagocytes. *Tmem268*^+/+^ and *Tmem268*^*−/−*^ mice were injected with LPS in the abdominal cavity for 2 h, followed by an additional injection of serum-opsonized Red Fluorescence Protein (RFP)–*Escherichia coli* for 30 min. Flow cytometry was used to analyze the phagocytosis by phagocytes in the abdominal cavity of *Tmem268*^+/+^ and *Tmem268*^*−/−*^ mice. As shown in Figs. EV4 and  3A, the proportion of RFP^+^F4/80^+^, RFP^+^Ly6C^+^, and RFP^+^Ly6G^+^ cells in *Tmem268*^*−/−*^ mice was significantly lower than that in *Tmem268*^*+/+*^ mice, indicating a lower level of phagocytosis of serum-opsonized *E.coli*. We performed similar experiments in *Tmem268*^*−/−*^ macrophages in vitro and found that the phagocytosis of serum-opsonized RFP-*E.coli* was inhibited in LPS-stimulated *Tmem268*^*−/−*^ macrophages (Fig. 3B,C,F).

**Figure EV4:**

*Tmem268* knockout impairs phagocytosis of serum-opsonized RFP-*E. coli.* (**A**–**C**) *Tmem268*^*+/+*^ and *Tmem268*^*−/−*^ mice (*n* = 5) were intraperitoneally injected with LPS for 2 h, following injected with serum-opsonized RFP-*E.coli* for 30 min. Flow cytometry analysis of RFP^+^F4/80^+^(**A**), RFP^+^Ly6C^+^ (**B**), and RFP^+^Ly6G^+^ (**C**) cells in the abdominal cavity.

It is well known that pathogens are captured by either complement or IgG in the serum. To determine whether *Tmem268* knockout inhibits CR3-mediated phagocytosis, RFP-*E. coli* were opsonized with heat-inactivated serum and incubated with bone marrow-derived macrophages (BMDMs). As expected, the phagocytosis index of *Tmem268*^+/+^ and *Tmem268*^*−/−*^ BMDMs was not significantly different (Fig. 3D,G). Moreover, we found that in the presence of RFP-*E. coli* opsonized by IgG-free serum, *Tmem268*^*−/−*^ BMDMs showed a lower phagocytosis index than *Tmem268*^*+/+*^ BMDMs (Fig. 3E,H). These results suggest that complement-mediated phagocytosis was decreased in *Tmem268*^*−/−*^ macrophages. In addition, *Tmem268* knockout did not affect scavenger receptor-dependent or mannose receptor-dependent phagocytosis (Appendix Fig. S4). Collectively, these findings suggest that *Tmem268* knockout impairs CR3-mediated phagocytosis.

### *Tmem268* deletion impairs adhesion and migration of phagocytes accompanied by CD11b downregulation

**Figure 4:**
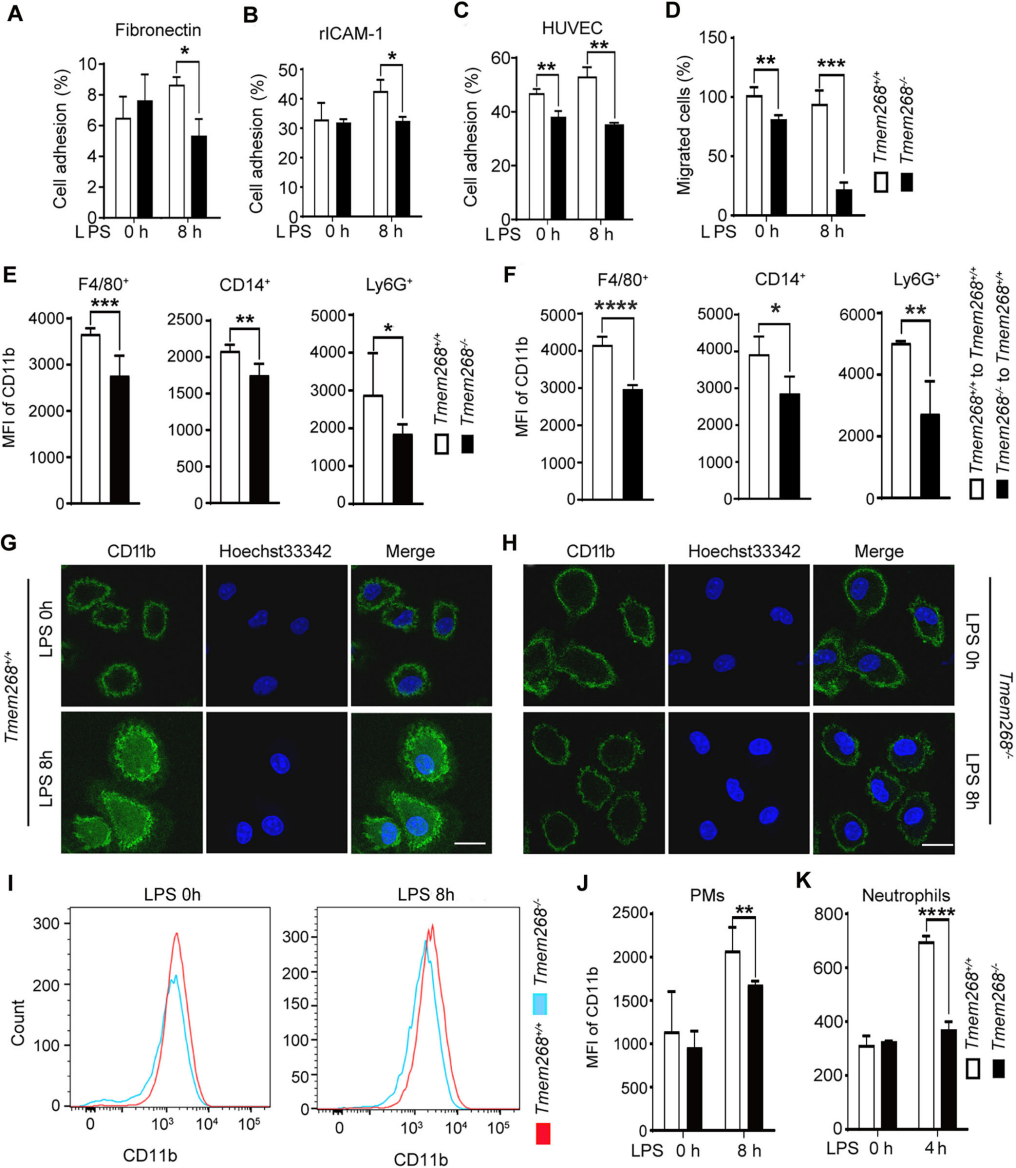
*Tmem268* deletion impairs adhesion and migration of phagocytes accompanied by the downregulation of CD11b expression. (**A**–**C**) *Tmem268*^*+/+*^ and *Tmem268*^*−/−*^ BMDMs (treated with or without LPS for 8 h) were allowed to attach to 96-well plates coated with (**A**) fibronectin, *P value = 0.0104, (**B**) recombinant ICAM-1, *P value = 0.0113, (**C**) HUVEC monolayer, for LPS 0 h, **P value = 0.0081, for LPS 8 h, **P value = 0.0014. Nonadherent cells were removed at 30 min, and the proportions of adhered cells were quantified using MTS assay. Unpaired two-tailed *t* test. Data (mean ± SD) are representative of at least three independent experiments. (**D**) Quantification of migrated *Tmem268*^*+/+*^ and *Tmem268*^*−/−*^ BMDMs (treated with or without LPS for 8 h) across HUVECs was determined by Transwell assay. For LPS 0 h, **P value = 0.0079, for LPS 8 h, ***P value = 0.0005. Unpaired two-tailed *t* test. Data (mean ± SD) are representative of at least three independent experiments. (**E**, **F**) The expression of CD11b of F4/80^+^, CD14^+^, and Ly6G^+^ phagocytes in the peritoneal cavity was analyzed by flow cytometry in different mice with CLP at 8 h. The mean fluorescence intensity (MFI) of CD11b in different group was statistically analyzed, respectively. *P < 0.05, **P < 0.01, ***P < 0.001. Unpaired two-tailed *t* test. Mean ± SD. *n* = 6 (**E**), *n* = 4 (**F**). (**G**, **H**) Representative fluorescence images of CD11b expression from *Tmem268*^*+/+*^ and *Tmem268*^*−/−*^ BMDMs treated with or without LPS for 8 h. Nuclei were stained with Hoechst 33342. Scale bars = 10 μm. (**I**) *Tmem268*^*+/+*^ and *Tmem268*^*−/−*^ PMs were treated with or without LPS for 8 h, the expression of CD11b was detected by flow cytometry. (**J**) The MFI of CD11b in *Tmem268*^*+/+*^ and *Tmem268*^*−/−*^ PMs was statistically analyzed. All plots represent mean ± SD from at least three independent experiments. Unpaired two-tailed *t* test. **P value = 0.0054. (**K**) The MFI of CD11b in *Tmem268*^*+/+*^ and *Tmem268*^*−/−*^ neutrophils was statistically analyzed. All plots represent mean ± SD from at least three independent experiments. ****P < 0.0001. Unpaired two-tailed *t* test. Source data are available online for this figure.

During infection, phagocyte recruitment from blood involves the rolling of the cells on the vascular endothelium, which is mediated by the interactions between selectins and their glycosylated ligands (Liew and Kubes, 2019). Then, the binding of integrins to ICAM-1 or VCAM-1 facilitates firm adhesion between phagocytes and endothelial cells and then promotes phagocyte transmigration by paracellular or transcellular routes (Liew and Kubes, 2019; Sun et al, 2020). In the present study, we investigated whether *Tmem268* ablation affects the adhesion and migration of phagocytes. As shown in Fig. 4A,B, compared with *Tmem268*^+/+^ BMDMs, *Tmem268*^*−/−*^ BMDMs showed impaired adhesion to fibronectin and recombinant ICAM-1 (rICAM-1) upon LPS stimulation. Crystal violet staining results were consistent with these findings (Appendix Fig. S5).

Because fibronectin and ICAM-1 are specific ligands for β2 integrins, we further evaluated the adhesion of BMDMs to human umbilical vein endothelial cells (HUVECs). As expected, the ability of *Tmem268*^*−/−*^ BMDMs adhering to HUVECs was significantly lower than that of *Tmem268*^+/+^ BMDMs (Fig. 4C). Subsequently, a Transwell assay was performed to explore the effect of TMEM268 on phagocyte migration. As shown in Fig. 4D, the transmigration of *Tmem268*^*−/−*^ BMDMs across HUVEC monolayers was significantly lower, especially in the presence of LPS. These results suggest that *Tmem268* inactivation inhibits the adhesion and migration of phagocytes.

As one of the key adhesion molecules expressed exclusively on immunocyte membranes, CD11b/CD18 participates in the regulation of leukocyte adhesion and migration and phagocytosis of pathogens (Huy et al, 2022; MacPherson et al, 2011). Our repeated flow cytometry analysis suggested that CD11b fluorescence intensity in macrophages, monocytes, and neutrophils was significantly lower in *Tmem268*^*−/−*^ CLP mice (Fig. 4E). Similar results were obtained in chimeric CLP mice reconstituted with *Tmem268*^*−/−*^ BM (*Tmem268*^*−/−*^ to *Tmem268*^*+/+*^; Fig. 4F), indicating a significant downregulation of CD11b protein in *Tmem268*-deficient phagocytes. Furthermore, confocal microscopy observations showed that LPS-stimulated *Tmem268*^*−/−*^ macrophages displayed weaker fluorescence signaling of CD11b than that in *Tmem268*^*+/+*^ macrophages (Fig. 4G,H). Data from flow cytometry analysis in peritoneal macrophages (PMs) were consistent with the results of confocal microscopy (Fig. 4I,J). Moreover, LPS-treated *Tmem268*^*−/−*^ neutrophils showed significantly lower CD11b expression than LPS-treated *Tmem268*^*+/+*^ neutrophils (Fig. 4K). In addition, qRT-PCR data showed that the levels of CD11b mRNA remained unchanged in *Tmem268*-deficient PMs (Appendix Fig. S6). Collectively, these results indicate that *Tmem268* knockout downregulates the membrane expression of CD11b protein.

Given that CD11b/CD18 is formed through the non-covalent association of α-subunit (CD11b) and β-subunit (CD18), we next analyzed whether TMEM268 affects the expression of CD18. As illustrated in Appendix Fig. S7A–D, flow cytometry and confocal microscopy detected no significant changes in CD18 expression between the *Tmem268*^*+/+*^ and *Tmem268*^*−/−*^ macrophages. In addition, results from flow cytometry suggested that TMEM268 did not affect the expression of CD11c in macrophages (Appendix Fig. S7E,F). Collectively, our findings suggest that *TMEM268* inactivation inhibits phagocyte adhesion, migration, and phagocytosis by downregulating the membrane expression of CD11b.

### *Tmem268* knockout promotes CD11b degradation via the endosomal–lysosomal pathway

**Figure 5:**
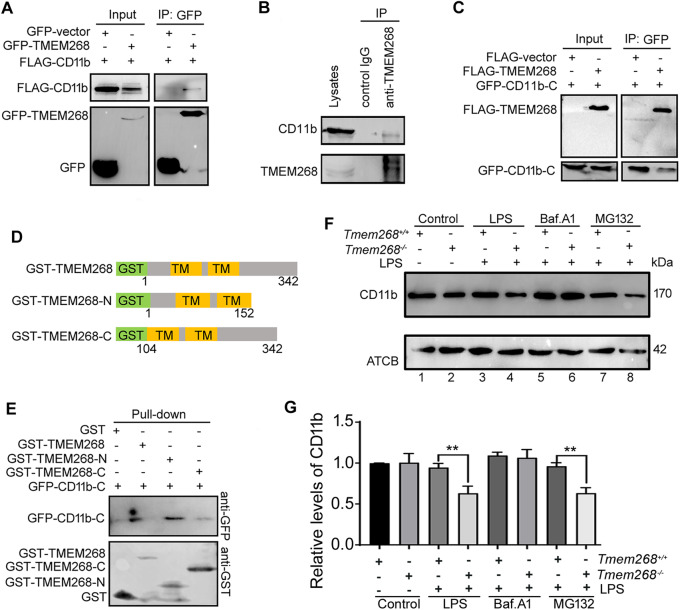
TMEM268 interacts with the C-terminus of CD11b via its N-terminus. (**A**) HEK293T cells were co-transfected with indicated plasmids for 24 h. Cell lysates were subjected to IP using an anti-GFP, and the indicated proteins were detected in the immunoprecipitates by western blotting. Simultaneously, 10% cell lysates were used to immunoblotting. (**B**) THP-1 cell lysates were subjected to IP using an anti-TMEM268 or IgG isotype control, and the indicated proteins were detected in the immunoprecipitates by western blotting. (**C**) HEK293T cells were co-transfected with indicated plasmids for 24 h. Cell lysates were subjected to IP using an anti-GFP, and the indicated proteins were detected in the immunoprecipitates by western blotting. (**D**) Construction of truncated TMEM268 prokaryotic plasmids. (**E**) Recombinant GST-TMEM268, GST-TMEM268-N or GST-TMEM268-C fusion protein and the GST protein were purified and immobilized on Glutathione-Sepharose beads, then incubated with HEK293T cell lysates containing GFP-CD11b-C. Proteins retained on Glutathione-Sepharose were then blotted using the indicated antibodies. (**F**) *Tmem268*^*+/+*^ and *Tmem268*^*−/−*^ BMDMs were treated with: LPS (1 μg/ml) 8 h, or BafA1 (20 nmol/l) 2 h+ LPS (1 μg/ml) 8 h, or MG132 (10 μmol/l) 6 h+ LPS (1 μg/ml) 8 h. The level of CD11b was measured by western blotting. ACTB was used as the loading control. (**G**) Quantification of amounts of indicated protein relative to ACTB in cells. Average value in *Tmem268*^*+/+*^ control was normalized as 1. All plots represent mean ± SD from at least three independent experiments. Unpaired two-tailed *t* test. **P < 0.01. Source data are available online for this figure.

Furthermore, we investigated the molecular mechanism by which *Tmem268* knockout downregulates CD11b expression. Co-immunoprecipitation (Co-IP) experiments showed that FLAG-CD11b protein was present in the GFP-TMEM268 immunoprecipitates (Fig. 5A). Similarly, the endogenous Co-IP assay also verified that TMEM268 coprecipitated with CD11b in THP-1 cells (Fig. 5B), indicating that the two proteins interacted in a complex in cells. Further Co-IP assay suggested that C-terminus of CD11b (CD11b_1105-1152_) was responsible for binding TMEM268 (Fig. 5C). Pull-down experiments showed that the interaction of TMEM268 with CD11b was dependent on the N-terminus of TMEM268 (amino acids 1–152; Fig. 5D,E). Together with the results of Fig. 4E–K, these data suggest that the TMEM268–CD11b interaction may inhibit CD11b degradation.

**Figure EV5:**
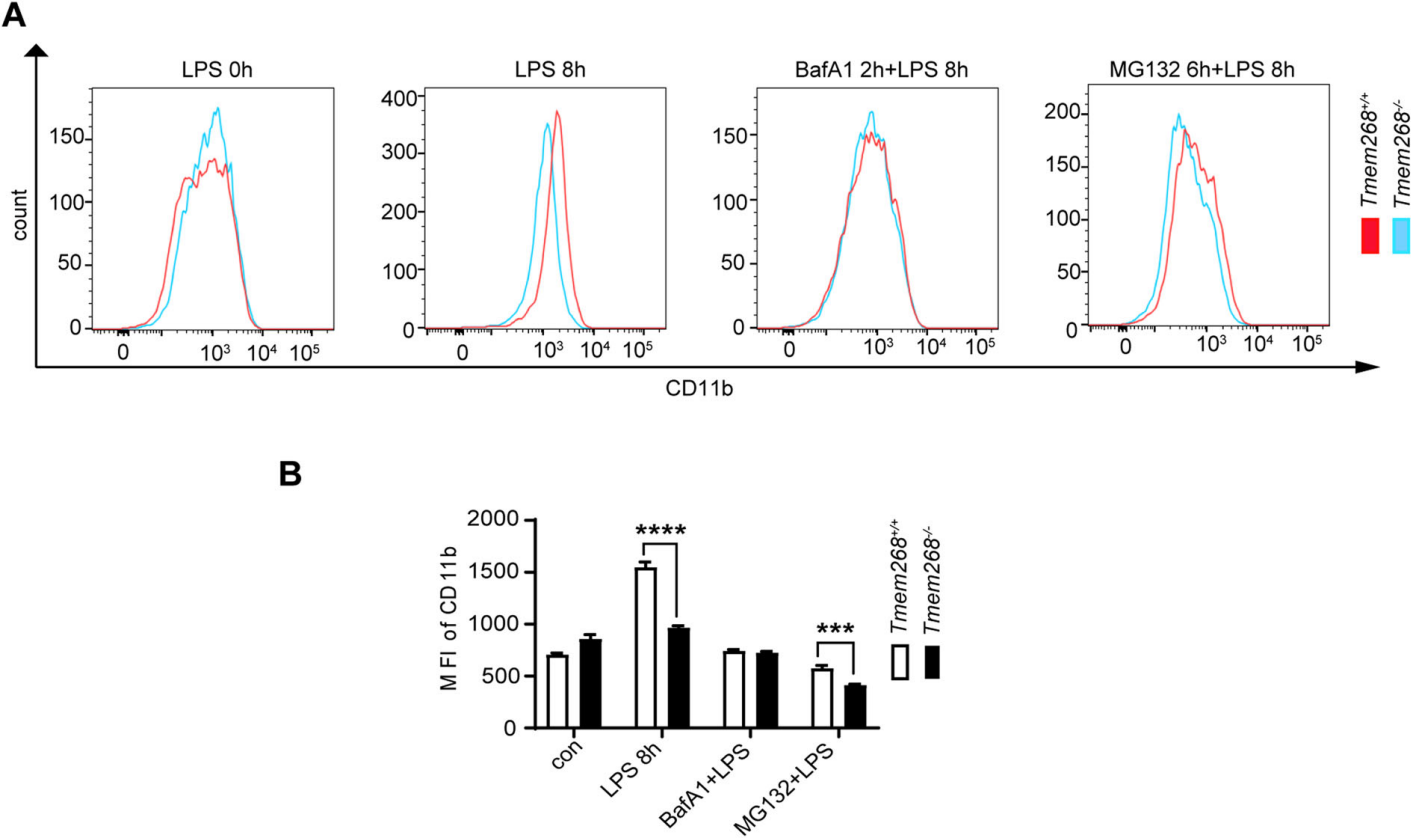
*Tmem268* knockout promotes CD11b degradation via the lysosomal pathway. (**A**) *Tmem268*^*+/+*^ and *Tmem268*^*−/−*^ BMDMs were treated as following: LPS (1 μg/ml) 8 h, or BafA1 (20 nmol/l) 2 h+ LPS (1 μg/ml) 8 h, or MG132 (10 μmol/l) 6 h+ LPS (1 μg/ml) 8 h. The membrane expression of CD11b was detected by flow cytometry. (**B**) The MFI of CD11b was statistically analyzed. For LPS 8 h, ****P < 0.0001, For MG132 + LPS, ***P value = 0.0007. Unpaired two-tailed *t* test. All plots represent mean ± SD from at least three independent experiments.

To determine whether CD11b degradation in *Tmem268*^*−/−*^ phagocytes is related to the proteasomal or lysosomal pathway, the proteasome inhibitor MG132 or the lysosome inhibitor bafilomycin A1 (BafA1) were added before LPS stimulation. Western blotting showed that the downregulation of CD11b protein (Fig. 5F, Lane 4 vs. Lane 3; Fig. 5G) in *Tmem268*^*−/−*^ BMDMs was significantly blocked by BafA1 treatment (Fig. 5F, Lane 6 vs. Lane 4; Fig. 5G) but not by MG132 treatment (Fig. 5F, Lane 8 vs. Lane 4; Fig. 5G). Similar results were obtained by flow cytometry (Fig. EV5), indicating that *Tmem268* knockout increased lysosomal degradation of CD11b protein.

**Figure 6:**
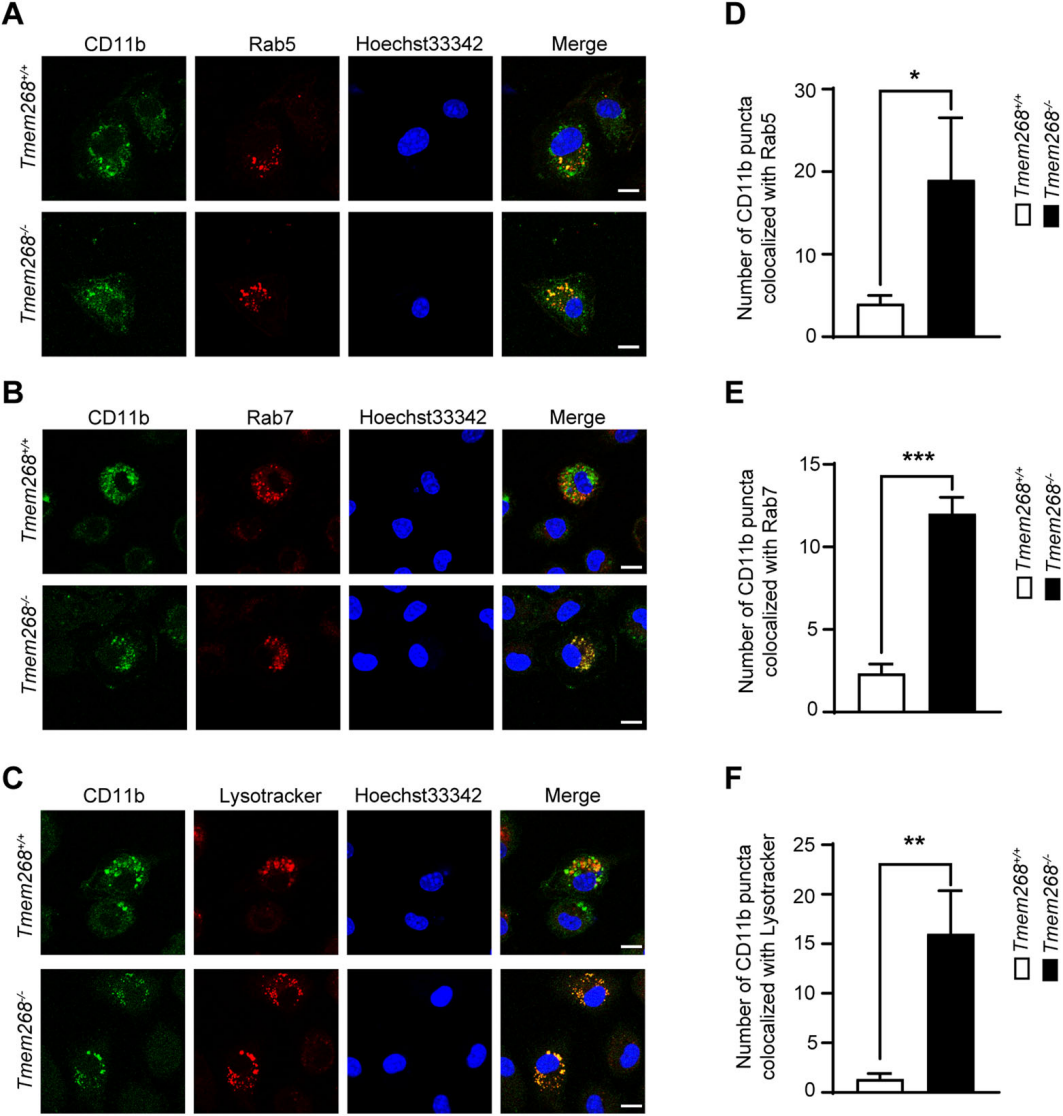
*Tmem268* knockout promotes CD11b degradation via the endosomal–lysosomal pathway. (**A**–**C**) *Tmem268*^*+/+*^ and *Tmem268*^*−/−*^ BMDMs were treated with LPS for 4 h, the co-localization of CD11b with (**A**) Rab5, or (**B**) Rab7 or (**C**) Lysotracker was observed by confocal microscope. Representative fluorescence images are shown. Scale bars = 10 μm. (**D**–**F**) The number of CD11b puncta colocalized with Rab5 (**D**), or Rab7 (**E**), or Lysotracker (**F**) was calculated. All plots represent mean ± SD from at least three independent experiments. Unpaired two-tailed *t* test. *P value = 0.027. **P value = 0.0045, ***P value = 0.0001. Source data are available online for this figure.

After internalization, integrin β2 is transferred to early endosomes, from where it is transported to late endosomes, which eventually undergo fusion with lysosomes (Moreno-Layseca et al, 2019). Confocal microscopy confirmed that in LPS-stimulated *Tmem268*^*−/−*^ BMDMs, co-localization of CD11b with early endosomes (Rab5; Fig. 6A,D), late endosomes (Rab7; Fig. 6B,E), and lysosomes (Lysotracker; Fig. 6C,F) was significantly higher than that in *Tmem268*^*+/+*^ BMDMs. Moreover, because integrin degradation is also connected to the autophagic machinery (Molnár et al, 2022), we next examined the localization of CD11b and LC3 in LPS-stimulated BMDMs. However, there was no significant difference between *Tmem268*^*+/+*^ and *Tmem268*^*−/−*^ groups (Appendix Fig. S8). These data suggest that *Tmem268* knockout promotes CD11b degradation via the endosomal–lysosomal pathway but not autophagy and that the TMEM268–CD11b interaction plays an important role in maintaining the protein homeostasis of CD11b.

### *Tmem268* knockout inhibits FAK/Src signaling pathway

**Figure 7:**
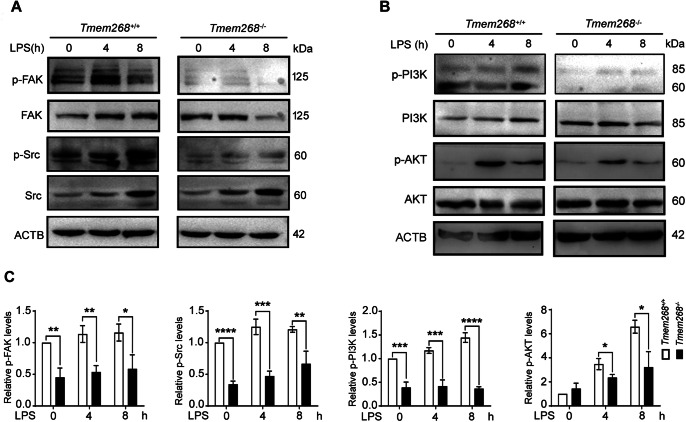
*Tmem268* knockout downregulates the CD11b/FAK/Src signaling pathway. (**A**, **B**) *Tmem268*^*+/+*^ and *Tmem268*^*−/−*^ BMDMs were treated with 1 μg/ml LPS for the indicated time. The levels of phospho-FAK, total FAK, phospho-Src, total Src, phospho-PI3K, total PI3K, phospho-AKT, total AKT were detected by western blot. ACTB was used as the loading control. (**C**) Quantification of amounts of indicated protein relative to ACTB in cells. The average value in *Tmem268*^*+/+*^ LPS 0 h was normalized as 1. All plots represent mean ± SD from at least three independent experiments. Unpaired two-tailed *t* test. *P < 0.05, **P < 0.01, ***P < 0.001, ****P < 0.0001. Source data are available online for this figure.

Integrins represent the key membrane receptors for sensing extracellular matrix ligands such as fibronectin, laminin, and collagen, triggering downstream pathways that determine cell adhesion and migration. The binding of β2 integrin and its ligands promotes the autophosphorylation of focal adhesion kinase (FAK), which leads to the formation of a dual-activated FAK–Src complex and results in the activation of PI3K/AKT pathway (Lorusso et al, 2020; Rose et al, 2007; Schaller et al, 1994). Therefore, we evaluated the proteins involved in the FAK/Src signaling pathway in *Tmem268*^*+/+*^ and *Tmem268*^*−/−*^ BMDMs. As shown in Fig. 7A–C, *Tmem268*^*−/−*^ BMDMs showed significantly lower levels of phosphorylated FAK, Src, PI3K, and AKT than *Tmem268*^*+/+*^ BMDMs, indicating that *Tmem268* deficiency inhibited the FAK/Src signaling pathway.

## Discussion

In this study, we investigated the role of TMEM268 in anti-infectious immune responses. Our findings demonstrate that *Tmem268* deletion impaired phagocyte adhesion, migration, and phagocytosis, which eventually inhibited phagocyte recruitment to the local infection site and impaired bacterial clearance.

β2 integrins are heterodimeric membrane receptors composed of a variable α subunit and a constant β subunit and are mainly expressed in leukocytes. The β2 integrin family includes LFA-1 (CD11a/CD18), Mac-1 (CD11b/CD18), αxβ2 (CD11c/CD18), and αdβ2 (CD11d/CD18) (Bednarczyk et al, 2020). Increasing evidence suggests that β2 integrins mediate various biological activities, including cell adhesion, migration, phagocytosis, proliferation, and survival (Yuki and Hou, 2020). Mac-1 (CD11b/CD18), an important member of the β2 integrin family, facilitates firm adhesion between phagocytes and endothelial cells during bacterial infection. Subsequently, phagocytes reach the site of infection through rolling on endothelial cells, intravascular crawling, and paracellular or transcellular transmigration. Our study demonstrated that *Tmem268* knockout downregulated the membrane expression of CD11b on phagocytes in CLP mice and on LPS-stimulated macrophages and neutrophils; furthermore, the adhesion of *Tmem268*^*−/−*^ phagocytes to fibronectin and rICAM-1 and their migration on HUVECs were impaired. Such effects may account for the diminished infiltration of phagocytes in CLP-induced sepsis. Moreover, the phagocyte dysfunction may also contribute to impaired host defense. CD11b/CD18, also known as complement receptor CR3, plays a key role in the uptake of iC3b-opsonized bacteria, apoptotic cells, and cell debris by phagocytes (Ehlers, 2000; Huang et al, 2015). The impaired phagocytosis of complement-opsonized *E.coli* owing to *Tmem268* deficiency was verified in vitro and in vivo, which suggests a positive role of TMEM268 in regulating complement-mediated phagocytosis. *Tmem268*^*−/−*^ mice were more susceptible to bacteria infection, aggravated cellular injury, and organ dysfunction, which ultimately resulted in higher mortality. Simultaneously, our data provided evidence for a positive regulatory role of TMEM268 in maintaining CD11b protein homeostasis. These findings provide valuable insights to understand the complex regulatory network of β2 integrins.

Mechanistically, the TMEM268–CD11b interaction may inhibit CD11b degradation via the endosomal–lysosomal pathway, which is crucial for its positive function in antibacterial defense. However, the intracellular trafficking of integrins is distinct and complex. Integrin receptors are initially endocytosed via multiple mechanisms, broadly classified as clathrin-mediated endocytosis (CME) and clathrin-independent endocytosis (Paul et al, 2015). After internalization, integrins are transported to Rab5-positive early endosomes, where the material is sorted according to whether the protein will undergo degradation or be recycled back to the plasma membrane (De Franceschi et al, 2015; Perini et al, 2014). Usually, early endosomes mature into late endosomes, which subsequently fuse with lysosomes to form endolysosomes. These biological events are finely regulated by various molecules: the small GTPase Rab34 inhibits the degradation of β3 integrins by specifically binding to its cytoplasmic tail, thus promoting migration, invasion, and adhesion of breast cancer cells (Sun et al, 2018). Another study has identified that the Golgi-localized, γ-ear-containing Arf-binding protein 2 (GGA2), which facilitates Rab13-dependent recycling of β1 integrins to the plasma membrane, is required for efficient migration and invasion of cancer cells (Sahgal et al, 2019). Further research is needed to elucidate the exact molecular mechanism by which TMEM268 regulates CD11b degradation.

In addition to their roles in leukocyte adhesion and phagocytosis, β2 integrins were also demonstrated to modulate immunocyte proliferation, differentiation, and apoptosis (Bose et al, 2014; Whitlock et al, 2000; Wu et al, 2004; Yan et al, 2004) and TLR signaling pathways (Han et al, 2010; Ling et al, 2014). Our study indicated that the increased levels of serum proinflammatory cytokines (TNF-α, IFN-β, and MCP-1) in *Tmem268*^*−/−*^ CLP mice are associated with the enhanced TLR signaling induced by regulation of CD11b expression. Further evidence is required to support this hypothesis. Moreover, it is worth investigating whether *Tmem268* deficiency promotes leukocyte apoptosis in a β2 integrin-dependent manner.

In conclusion, our study demonstrates that TMEM268 deficiency inhibits phagocyte adhesion and migration and phagocytosis of bacteria, eventually leading to impaired pathogen elimination during sepsis. Therefore, the highly expressed TMEM268 in monocytes/macrophages may have a protective effect during infection. Our findings offer novel insights into the complex regulatory network of β2 integrins and provide a potentially promising approach to the treatment of sepsis and other related immune disorders.

## Methods

### Antibodies and reagents

Antibodies and reagents in this study are listed in Appendix Tables S1 and S2.

### Plasmid construction

FLAG-CD11b plasmid was purchased from Fenghuibio, Shanghai, China. The C-terminus of CD11b (CD11b_1105-1152_) cDNA was amplified from FLAG-CD11b by PCR using the forward primer (5′-CCAAGCTTCCCCTGCCGCTCATCGTGG-3′) and reverse primer (5′- CGGAATTCCTACTGGGGTTCGGC-3′), then cloned into pEGFP-C1-vector to construct the GFP-CD11b-C plasmid. GFP-TMEM268, FLAG-TMEM268, GST-TMEM268_1-342_, GST-TMEM268-N_1-152_, GST-TMEM268-C_104-342_ were previously constructed by our laboratory (Hong et al, 2019). All plasmids were confirmed by DNA sequencing.

### *Tmem268* gene KO mice

*Tmem268* KO mice of C57BL/6 background were produced using CRISPR/Cas9 genome editing with guide RNA (sgRNA1: 5′-GAGCCTCCCACAGATCCTGGTGG-3′, sgRNA2: 5′-TCCTGGCTGGGGGCAAGGTAAGG-3′) targeting exon 3 of mouse *Tmem268* at Shanghai BRL Medicine Inc. Offspring from the founder containing 85 base pairs (bp) deletion genotyping was performed by PCR using oligonucleotides 5ʹ-ATCGGAAGGTCAGCATTTA-3ʹ (forward) and 5ʹ-TAGGCAGTGGCAGTCAAGC-3ʹ(reverse) [wild-type (WT) allele (503 bp), mutant allele (416 bp)].

The mutant mice appeared phenotypically normal, and no obvious developmental and reproductive defects were observed. All mice were housed in a specific pathogen free facility at a constant room temperature with free access to water and standard mouse chow. All animal experimental procedures and techniques were approved by the Animal Ethics Committee of Peking University Health Sciences Center (LA2022406).

### Animal models

Male mice (aged 8–12 weeks) were intraperitoneally injected with LPS (15 mg/kg) to induce sepsis. Control mice received the same volume of PBS.

For cecal ligation and puncture model, male mice (aged 8 weeks) were intraperitoneally anesthetized with a combination of ketamine (100 mg/kg) and xylazinethe (7.5 mg/kg). The cecum was exposed under sterile surgical conditions and ligated at the distal 50% position. Then, the ligated cecum was punctured by a 21 G needle, and a small amount (droplet) of feces was gently extruded from the holes. The cecum was replaced into the peritoneal cavity and the abdomen was closed. The mice were housed in microisolators after surgery.

### Histopathological analysis

Mouse lung and kidney tissues were collected 24 h after the CLP surgery, fixed overnight in 4% paraformaldehyde, dehydrated in a graded series of ethanol, embedded in paraffin, and sliced into sections (4 μm). These sections were stained with hematoxylin and eosin (H&E) using standard procedures.

TUNEL assays were performed using an in situ cell death detection kit (Roche Applied Science, Indianapolis, IN, USA) according to the manufacturer’s instructions. The sections were counterstained with Hoechst 33342 (Sigma Aldrich, 14533).

### Bacterial counts

To determine the bacterial burden, whole blood, peritoneal lavage fluid, and tissue samples from mice were harvested 24 h after the CLP surgery. Equal weight of tissues were homogenized in sterile phosphate-buffered saline (PBS). All samples were serially diluted by sterile PBS and plated on Trypticase Soy Agar (TSA) plates. After incubated at 37 °C for 16–24 h, the number of bacterial colonies were calculated as colony forming units (CFU) for statistical analysis.

### Flow cytometry analysis

Single-cell suspensions were obtained from peritoneal lavage fluid, peripheral blood, bone marrow, spleen, lymph node, thymus and liver of male or female *Tmem268*^*+/+*^ and *Tmem268*^*−/−*^ mice. Then different cells were stained with fluorescently labeled antibodies, incubated at 4 °C for 30 min and analyzed by flow cytometry (FACS verse, BD Biosciences, San Jose, CA, USA).

### Cytokine detection

The levels of TNF-α, IL-6, IFN-β and MCP-1/CCL2 in mouse serum were measured by LEGENDplex™ mouse proinflammatory chemokine panel (740451; BioLegend, San Diego, CA, USA), according to the manufacturer’s instructions.

### Bone marrow transplantations

The recipients, male *Tmem268*^*+/+*^ and *Tmem268*^*−/−*^ mice (aged 6 weeks) were fed with acidified water (pH 2.5–3) contained neomycin (100 mg/l) and polymyxin B sulfate (60,000 U/l) 7 days before bone marrow transfer. After that, the recipient’s mice were lethally irradiated with a single dose of 10 Gy. 4 h later, freshly isolated bone marrow cells (1 × 10^7^ cells) from *Tmem268*^*+/+*^ or *Tmem268*^*−/−*^ mice were injected intravenously into the irradiated *Tmem268*^*+/+*^ and *Tmem268*^*−/−*^ mice to create bone marrow chimeras. The chimeras could recover under sterile conditions for 8 weeks.

### Cell isolation, culture and transfection

Mouse peritoneal macrophages (PMs) were collected from male or female mice intraperitoneally injected with 1–2 ml of 4% thioglycolate medium 3 days before euthanized, and cultured in Dulbecco’s Modified Eagle’s Medium (DMEM) with 10% fetal bovine serum (FBS).

Mouse bone marrow-derived macrophages (BMDMs) were prepared according to a published protocol with modifications (Toda et al, 2021). Briefly, bone marrow cells were isolated from mouse hind legs in single-cell suspension and cultured in DMEM supplemented with macrophage colony-stimulating factor (M-CSF) and 10% FBS for 7 days. BMDMs were harvested with ice-cold TEN buffer (40 mM Tris, 4 mM EDTA, 0.15 M NaCl, pH 8.0) and resuspended in DMEM with 10% FBS.

Mouse neutrophils were collected from male or female mice intraperitoneally injected with 1–2 ml of 4% thioglycolate medium 8–12 h before euthanized, and cultured in RPMI 1640 Medium supplemented with 0.5% FBS.

Mouse bone marrow-dendritic cells (BMDCs) were obtained from culturing of bone marrow cells of male or female mice in DMEM with M-CSF (50 ng/ml) plus IL-4 (1 ng/ml) for 5 days. THP-1 were cultured in RPMI 1640 Medium supplemented with 10% FBS. Human Umbilical Vein Endothelial Cells (HUVECs) were cultured in Endothelial Cell Medium (ECM) with 1% endothelial cell growth factor and 10% FBS. HEK293T cell lines were cultured in DMEM with 10% FBS. Cells were transfected with plasmids using NEOFECT Reagent according to the manufacturer’s instructions.

### Phagocytosis assays

*Tmem268*^*+/+*^ and *Tmem268*^*−/−*^ mice (male, aged 6–8 weeks) were intraperitoneally injected with LPS (5 mg/kg) for 2 h, then 1 × 10^8^ serum-opsonized RFP-*E. coli* (incubated with 50% serum at 37 °C for 1 h) were injected into the peritoneum. 30 min after the injection, the peritoneal cells were harvested by collecting the peritoneal lavage fluid, following labeled with fluorescein-conjugated anti-F4/80, anti-Ly6C, anti-Ly6G antibodies. The phagocytic efficiency of RFP-*E. coli* was analyzed by flow cytometry (FACS verse, BD Biosciences, San Jose, CA, USA).

BMDMs were pre-treated with LPS (1 μg/ml) for 8 h and then incubated with RFP-*E.coli* or serum-opsonized RFP-*E. coli* at a ratio of 50:1 cells for 30 min. Complement-free serum was prepared by heating at 56 °C for 30 min, while IgG-free serum was prepared by incubating with Protein A overnight at 4 °C. BMDMs were washed three times with cold PBS, and the phagocytosis was observed by confocal microscopy (Zeiss LSM880, Oberkochen, Germany).

### Immunofluorescence and confocal microscopy assays

Cells were cultured on the glass coverslips in 24-well plates. For membrane protein detection, cells were incubated directly with the primary antibodies at 4 °C for 30 min. After washing the plates for three times with PBS, FITC/TRITC-conjugated secondary antibodies were added and incubated with cells for 30 min at 4 °C, followed by cell fixation using 4% paraformaldehyde. For intracellular protein detection, cells were fixed with 4% paraformaldehyde, permeabilized with 0.2% Triton X-100 and blocked with 5% bovine serum albumin (BSA) in PBS. These cells were then incubated with primary antibodies overnight at 4 °C, stained with FITC/TRITC-conjugated secondary antibodies and imaged by a Zeiss LSM880 Confocal Microscope.

### Cell adhesion assays

The 96-well plates were coated with fibronectin (10 μg/ml) or recombinant intercellular adhesion molecule 1 (rICAM-1) (5 μg/ml) overnight at 4 °C. Next, nonspecific binding sites were blocked with 1% (BSA) in PBS for 30 min at room temperature. BMDMs treated with or without LPS (1 μg/ml) for 8 h were added into the ligand-coated wells (5 × 10^4^ cells/well) and incubated at 37 °C for 30 min. Nonadherent cells were removed by washing with DMEM for three times. Cell adhesion was quantified using MTS according to the manufacturer’s instructions, and the optical density was measured at 490 nm. The attached cells were also fixed with methanol and stained with crystal violet for 15 min.

HUVECs were seeded on 96-well plates (5 × 10^4^ cells/well) and stimulated with LPS (100 ng/ml) for 8 h to promote the membrane expression of ICAM-1. BMDMs (2 × 10^5^/well) cells treated with or without LPS (1 μg/ml) were then added on confluent HUVEC monolayer and incubated for 30 min at 37 °C. Nonadherent cells were gently washed off and the attached cells were analyzed by MTS assay. Wells containing only HUVECs were used to determine background value.

### Transmigration assays

To evaluate phagocytes transmigration across the HUVEC monolayer, HUVECs (5 × 10^4^ cells/well) were seeded into the upper chamber of 8-μm-pore filter Transwell plates and treated with LPS (100 ng/ml) for 8 h to promote the membrane expression of ICAM-1. After removing the medium in upper chamber, LPS (1 μg/ml) pre-treated BMDMs (in 200 μl serum-free DMEM) were added, and 500 μl DMEM with 10% FBS was added into the lower chamber. BMDMs were allowed to migrate through the HUVEC monolayer into the lower chamber at 37 °C for 2 h, transmigration was stopped by carefully removing the upper chamber. Filters were immersed in methanol for 5 min and then stained with crystal violet for 15 min. The cells on the filters were counted under Olympus DP72 microscope.

### Reverse-transcription (RT)-PCR and quantitative real-time (qRT)-PCR assays

Total RNA samples were extracted from cells with the TRIzol reagent. In total, 1 μg acquired RNA was reversely transcribed into cDNA using RevertAid First Strand cDNA Synthesis Kit according to the manufacturer’s instructions. RT-PCR was performed using the ThermoScript RT-PCR System. Quantitative real-time PCR was performed using SYBR Green qPCR Mix. The primers against the indicated genes used in this study are listed in Appendix Table S3. All mouse genes expression was normalized to β-actin (ACTB).

### Co-immunoprecipitation and western blot analysis

For the IP analysis, cells were collected and disrupted in IP lysis buffer with protease inhibitor cocktail (Roche Diagnostics, Berlin, Germany). Total cell extracts were incubated with precleared protein G Sepharose^TM^ Fast Flow and corresponding antibodies overnight at 4 °C. The beads were collected by centrifugation, washed three times and resuspended in 2×SDS loading buffer, subsequently analyzed by western blotting.

For normal Western blot analysis, protein concentrations were determined using a BCA protein assay reagent (Beyotime, Shanghai, China; P0010). Equal amounts of proteins were separated by SDS-PAGE electrophoresis and transferred to polyvinylirdenediflouride (PVDF) membranes (Millipore, USA). The membranes were blocked in 5% skimmed milk and incubated with corresponding primary and secondary antibodies. The membranes were then washed, and the protein was visualized with enhanced chemiluminescence solution and taken image using a chemiluminescent imaging system (iBright 750, Thermo Scientific, Waltham, MA, USA). The scanned bands were quantified using ImageJ software. The results were representative of at least three experiments.

### In vitro GST-pulldown assays

Soluble recombinant GST, GST-TMEM268, or GST-TMEM268 mutants were expressed in *Escherichia coli* strain BL21 (DE3) and purified. Equal amounts of these proteins immobilized on glutathione-Sepharose^TM^ 4B were incubated with whole cell lysates extracted from the indicated plasmids transfected HEK293T cells for 2 h at 4 °C. After five washes, the beads were resuspended in 2×SDS loading buffer and analyzed by western blotting.

### Statistical analysis

Data were expressed as mean ± experimental standard deviations (SD). Unpaired Student’s *t* test (two-tailed) was performed to assess the statistical significance between groups (significance: *P < 0.05; **P < 0.01; ***P < 0.001, ****P < 0.001). Survival curves were prepared using the Kaplan–Meier curve. All analyses were performed using GraphPad Prism 9.0.

### Graphics

Synopsis image was generated by the BioRender (https://www.biorender.com/).

## Supplementary information

Appendix

Source data Fig. 1

Source data Fig. 2

Source data Fig. 3

Source data Fig. 4

Source data Fig. 5

Source data Fig. 6

Source data Fig. 7

Peer Review File

Expanded View Figures

## Data Availability

The raw data of the flow cytometry screen are available in FlowRepository with the Repository ID FR-FCM-Z77N (http://flowrepository.org/id/FR-FCM-Z77N). The source data of this paper are collected in the following database record: biostudies:S-SCDT-10_1038-S44319-024-00141-6.
