# Supplementary material for: Deletion of Tmem268 in mice suppresses anti-infectious immune responses by downregulating CD11b signaling
Source: EMBO Rep. 2024 May 10;25(6):7. doi: 10.1038/s44319-024-00141-6 (PMC11169502; doi:10.1038/s44319-024-00141-6)
Supplement: Supplementary file 1 — Appendix [file 44319_2024_141_MOESM1_ESM.pdf]

Appendix for

Deletion of *Tmem268* suppresses anti-infectious immune responses by downregulating CD11b signaling

Table of Contents:

Appendix Figure S1, page 2
Appendix Figure S2, page 3
Appendix Figure S3, page 4
Appendix Figure S4, page 5
Appendix Figure S5, page 6
Appendix Figure S6, page 7
Appendix Figure S7, page 8 - 9
Appendix Figure S8, page 10
Appendix Table S1. List of antibodies used in this study, page 11 - 12
Appendix Table S2. List of reagents used in this study, page 13 - 14
Appendix Table S3. The qRT-PCR and genome PCR Primer sequences used in this study, page 15

Appendix Figure S1

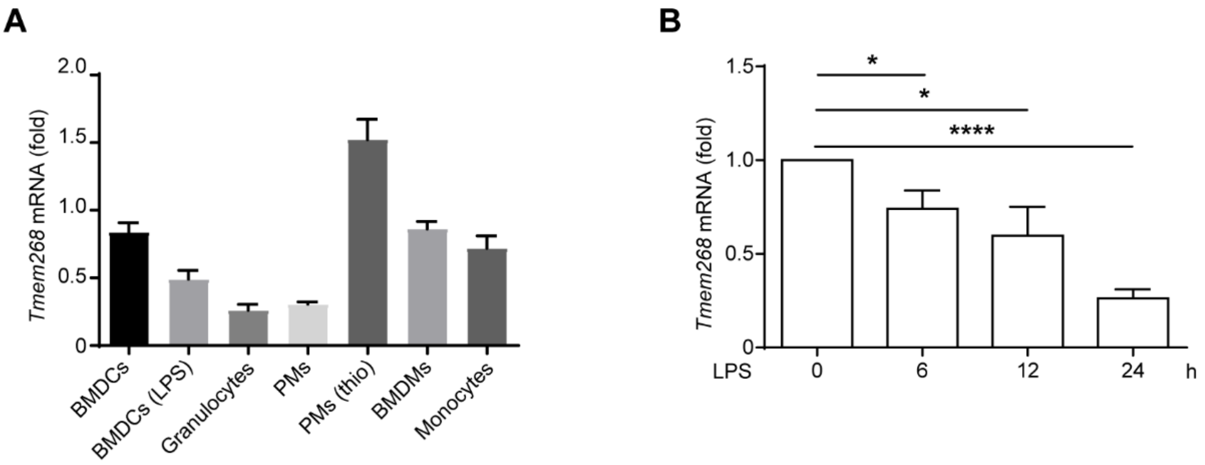

Appendix Figure S1. TMEM268 is highly expressed in monocytes/macrophages.

A qRT–PCR analysis of *Tmem268* mRNA levels in bone marrow-dendritic cells (BMDCs), granulocytes, peritoneal macrophages (PMs), bone marrow-derived macrophages (BMDMs) and monocytes of wild-type mice. PMs (thio): peritoneal macrophages collected from mice intraperitoneally injected with 4% thioglycolate (thio) medium. Mean ± SD (n=3).

B qRT–PCR analysis of *Tmem268* mRNA levels in PMs treated with 100 ng/ml LPS for indicated time. All plots represent mean ± SD from at least three independent experiments. Unpaired two-tailed *t*-test. For LPS 6 h versus LPS 0 h, **P* value=0.0102, for LPS 12 h versus LPS 0 h, **P* value=0.0108, for LPS 24 h versus LPS 0 h, *****P* <0.0001.

Appendix Figure S2

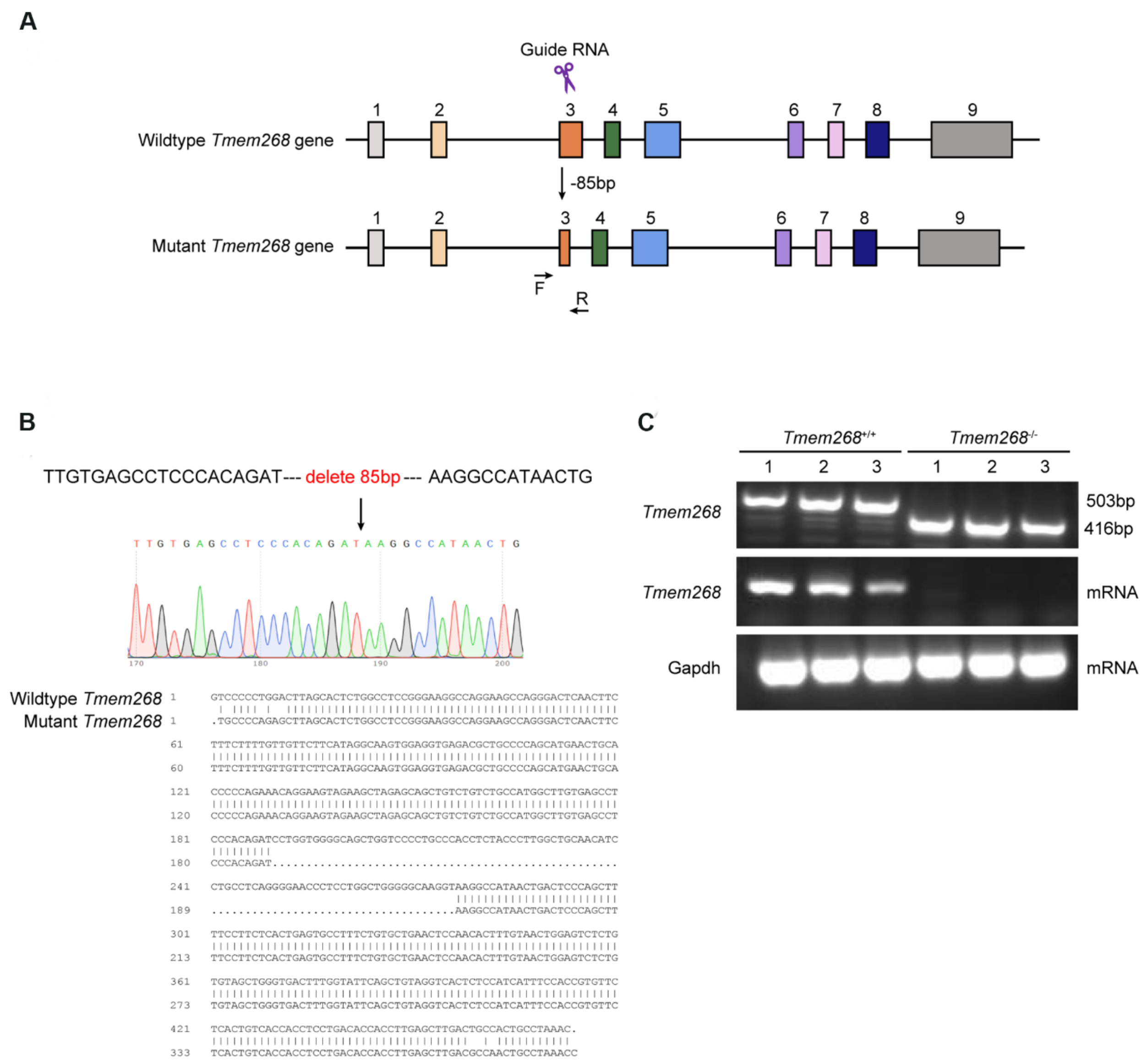

Appendix Figure S2. CRISPR/Cas9 genome editing of mouse *Tmem268*.

A The genomic structure of wild-type and mutant *Tmem268* gene is shown. The boxes represent exons of *Tmem268*, and the targeting sites are shown.

B Gene sequencing and analysis of wild-type and *Tmem268* knockout mice.

C Genomic PCR and RT-PCR were used to identify the mutations of *Tmem268* gene in mice.

Appendix Figure S3

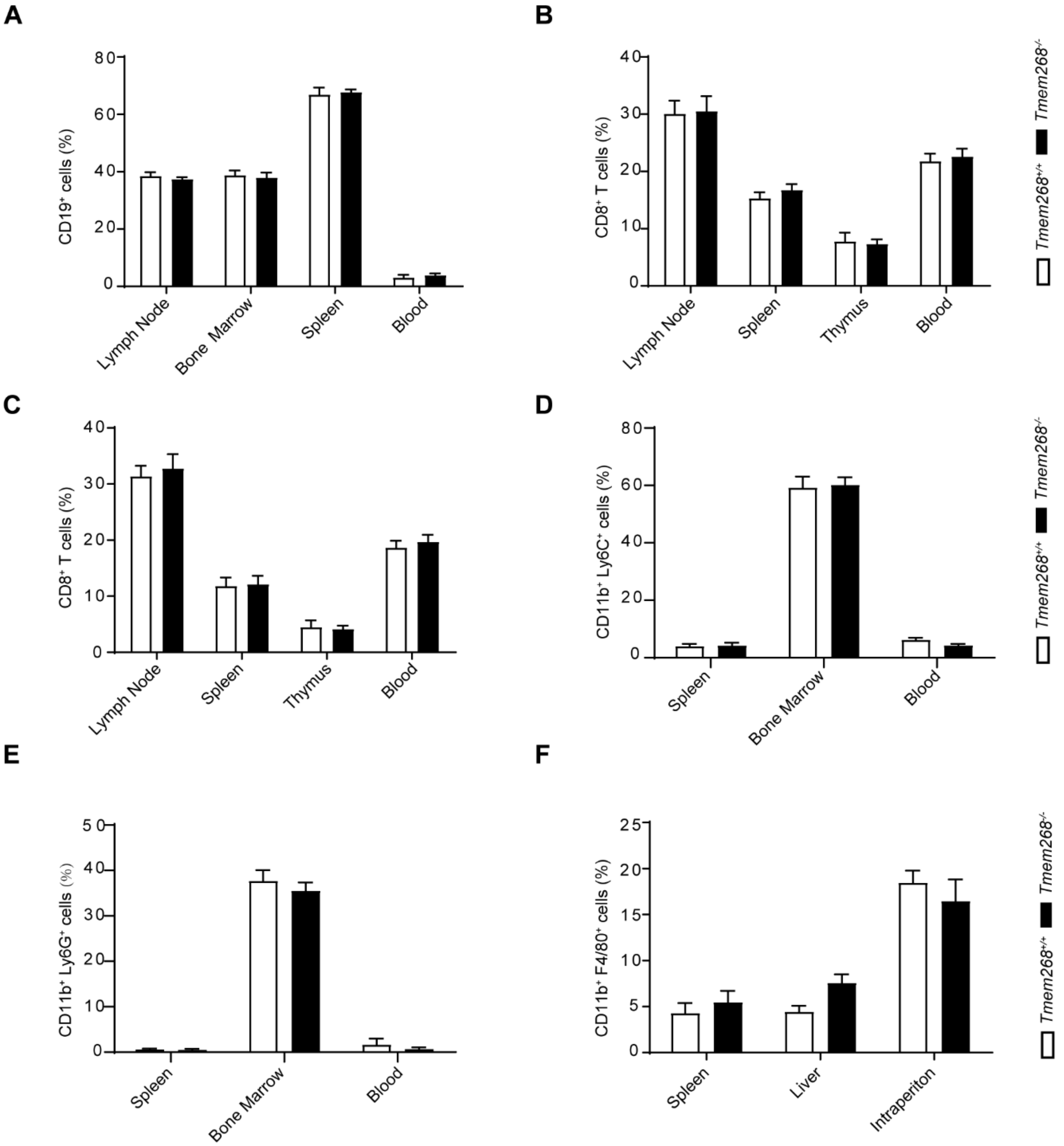

Appendix Figure S3. *Tmem268*-deficiency fails to affect the development of immunocytes.

A-F Flow cytometry analysis of the proportions of (A) $CD45^+CD19^+$ B cells, (B) $CD45^+CD4^+$ T cells, (C) $CD45^+CD8^+$ T cells, (D) $CD45^+CD11b^+Ly6C^+$ monocytes, (E) $CD45^+CD11b^+Ly6G^+$ neutrophils, (F) $CD45^+CD11b^+F4/80^+$ macrophages in the indicated tissues of $Tmem268^{+/+}$ and $Tmem268^{-/-}$ mice. Mean ± SD (n=3). Unpaired two-tailed *t*-test.

Appendix Figure S4

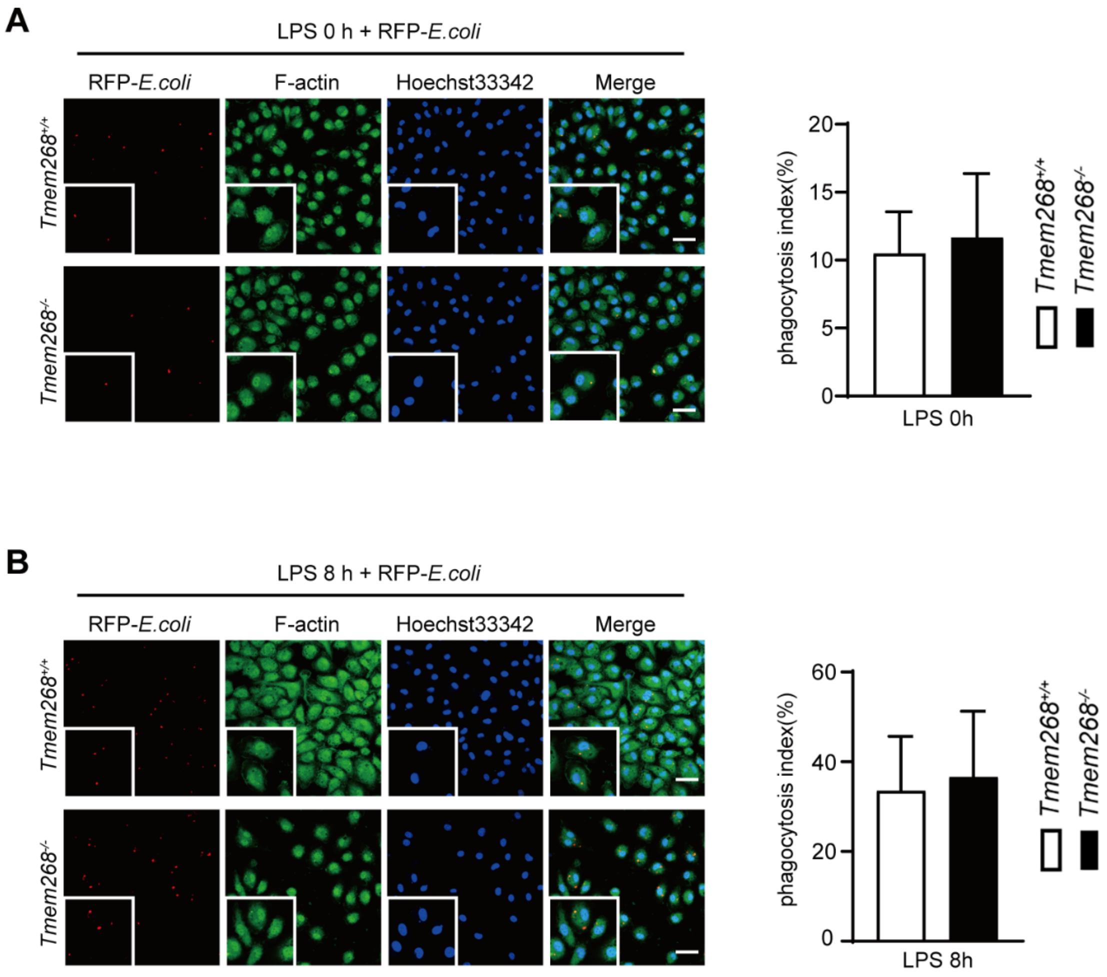

Appendix Figure S4. *Tmem268* knockout did not affect phagocytosis of unopsonized RFP-*E.coli*

A, B *Tmem268*$^{+/+}$ and *Tmem268*$^{-/-}$ BMDMs were treated without or with LPS for 8 h, then cultured with RFP-*E.coli* for 30 min. The representative fluorescence images and corresponding phagocytosis index of BMDMs are shown. Scale bars=20 μm. All plots represent mean ± SD from at least three independent experiments. Unpaired two-tailed *t*-test.

Appendix Figure S5

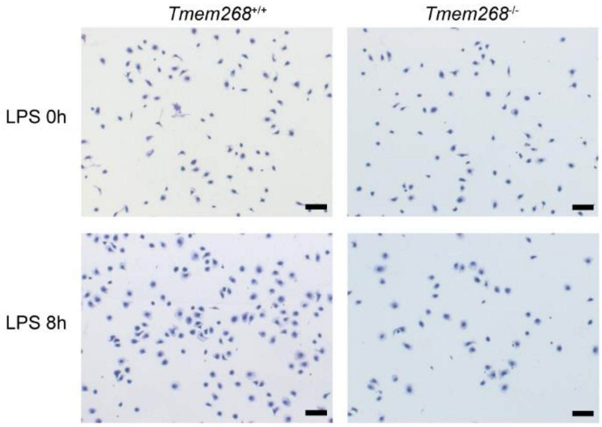

Appendix Figure S5. *Tmem268* deletion impairs macrophages adhesion.

Tmem268$^{+/+}$ and *Tmem268*$^{-/-}$ BMDMs (treated with or without LPS for 8 h) were added into rICAM-1-coated 96-well plates. 30 min later, nonadherent cells were washed and adhered cells were stained with crystal violet. Representative images of adhered cells are shown. Scale bars=100 μm.

Appendix Figure S6

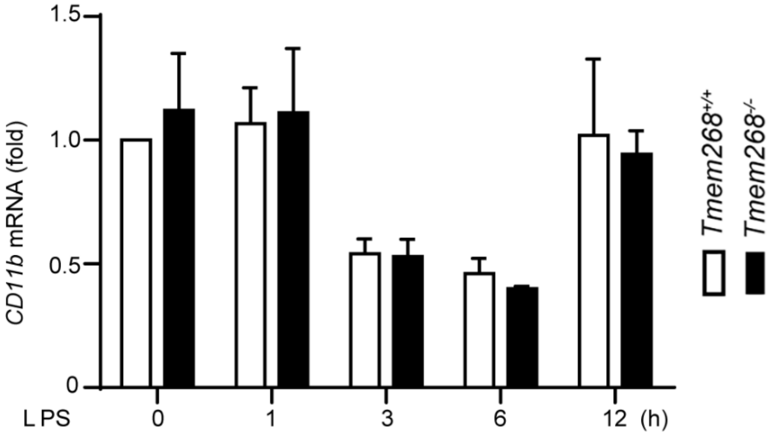

Appendix Figure S6. *Tmem268* knockout does not affect the levels of *CD11b* mRNA. qRT–PCR analysis of *CD11b* mRNA levels in PMs treated with LPS (100 ng/ml) for indicated time. All plots represent mean ± SD from at least three independent experiments. Unpaired two-tailed *t*-test.

Appendix Figure S7

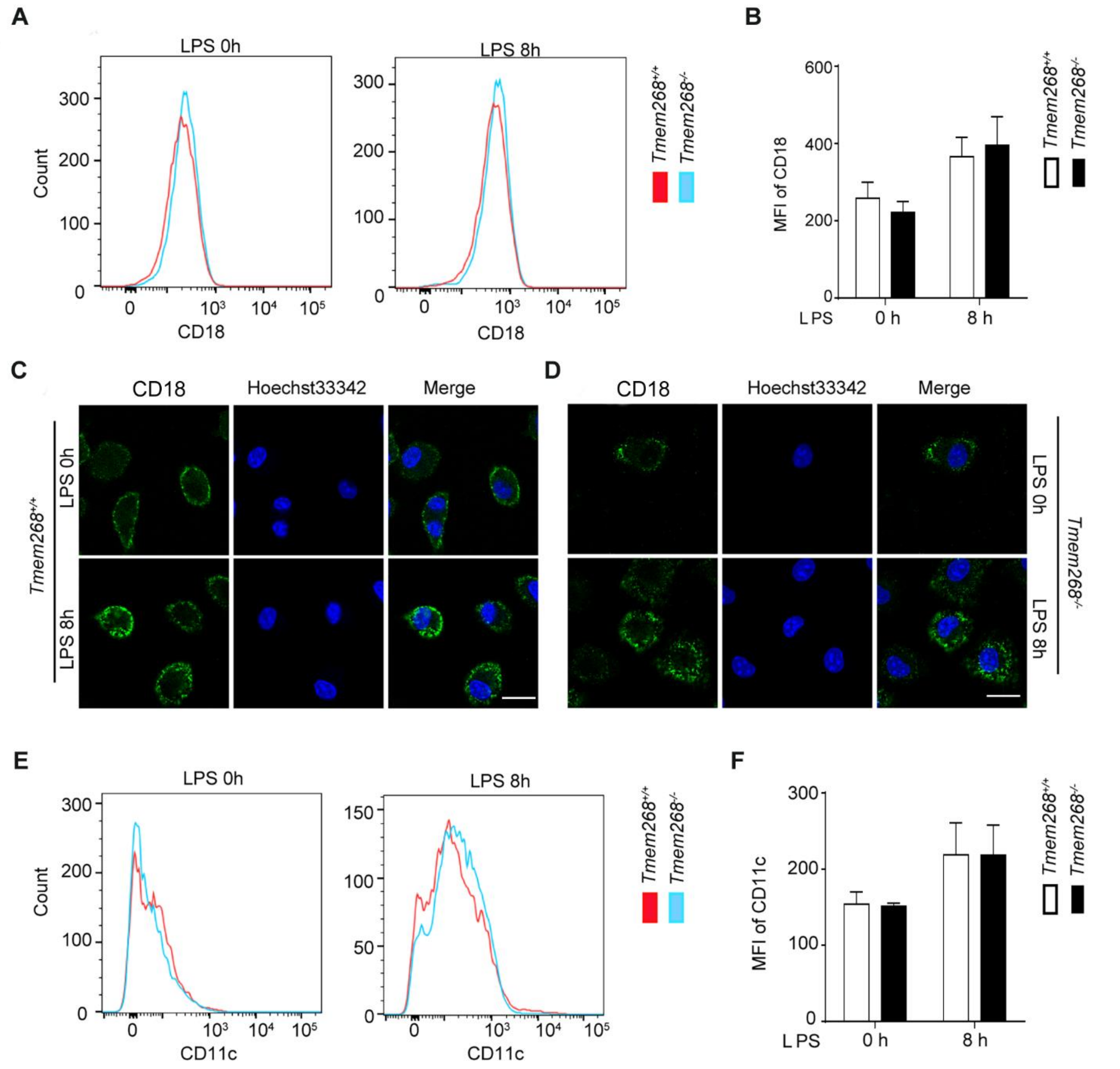

Appendix Figure S7. *Tmem268* knockout does not affect CD18 and CD11c expression.

A *Tmem268*$^{+/+}$ and *Tmem268*$^{-/-}$ BMDMs were treated with or without LPS (1 μg/ml) for 8 h, the membrane expression of CD18 was detected by flow cytometry.

B The MFI of CD18 was statistically analyzed. All plots represent mean ± SD from at least three independent experiments. Unpaired two-tailed *t*-test.

C, D Representative fluorescence images of CD18 (green) from *Tmem268*$^{+/+}$ and *Tmem268*$^{-/-}$ BMDMs treated with or without LPS for 8 h. Nuclei were stained with Hoechst33342. Scale bars=10 μm.

E *Tmem268*$^{+/+}$ and *Tmem268*$^{-/-}$ PMs were treated with or without LPS (1 μg/ml) for 8 h, the membrane expression of CD11c was detected by flow cytometry.

F The MFI of CD11c was statistically analyzed. All plots represent mean ± SD from at least three independent experiments. Unpaired two-tailed *t*-test.

Appendix Figure S8

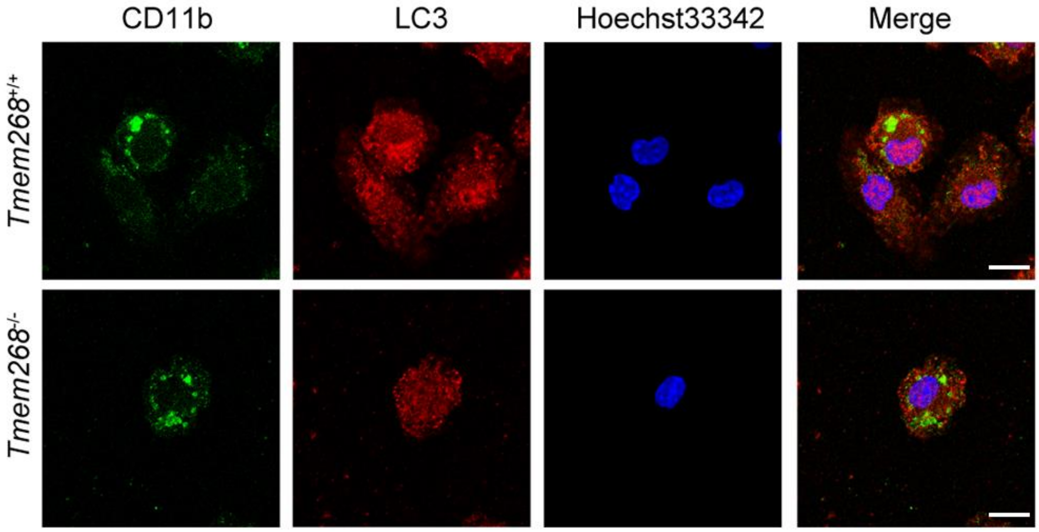

Appendix Figure S8. *Tmem268* knockout does not affect the colocalization of CD11b with LC3.

Tmem268$^{+/+}$ and *Tmem268*$^{-/-}$ BMDMs were treated with LPS (1 μg/ml) for 4 h, then performed with immunofluorescence assay. Nuclei were stained with Hoechst33342. Scale bars=10 μm. Representative confocal images are shown.

Appendix Table S1. List of antibodies used in this study

Antibodies	No. of Product	Company
APC anti-mouse CD4	553051	
APC anti-mouse Ly6G	127613	
FITC anti-mouse CD45	103107	
FITC anti-mouse CD18	101405	
PE anti-mouse CD14	562691	
PE anti-mouse F4/80	123109	Biolegend Company, San Diego, CA, USA
PE anti-mouse CD8a	100707	
PE-Cy7 anti-mouse Ly6C	128017	
PE-Cy7 anti-mouse CD19	552854	
PerCP anti-mouse/human CD11b	101229	
Ultra-LEAF™ Purified anti-mouse Ly-6G Antibody	127649	
FAK Antibody Sampler Kit	9330	
Phospho-Src Family (Tyr416) Rabbit mAb	6943	
Src (32G6) Rabbit mAb	2123	
Phospho-PI3 Kinase p85 (Tyr458)/p55 (Tyr199) Antibody	4228	Cell Signaling Technology, Beverly, MA, USA
PI3 Kinase p85 Antibody	4292	
Phospho-Akt (Ser473) (D9E) XP® Rabbit mAb	4060	
Akt Antibody	9272	
Rab5A (E6N8S) Mouse mAb	46449	
Rab7 (D95F2) XP® Rabbit mAb	9367	
Anti-CD11b antibody [EPR1344]	ab133357	Abcam, Cambridge, England
CD11b Monoclonal Antibody (M1/70), eBioscience™	14-0112-82	Thermo Fisher Scientific, Waltham, MA, USA
Integrin β2/ITGB2/CD18 Antibody (CTB104)	sc-8420	Santa Cruz Biotechnology, Dallas, Texas, USA
Anti-LC3B antibody produced in rabbit	L7543	
Goat Anti-Rabbit IgG (H+L) HRP	AP307P	Sigma, St. Louis, MO, USA
Goat Anti-Mouse IgG (H+L) HRP	12-349	
Anti GFP-tag mouse monoclonal antibody	KM8009	

Anti GST-tag mouse monoclonal antibody	KM8005	Sungene Biotech Company, Tianjin, China
Anti Flag-tag mouse monoclonal antibody	KM8002	
Fluorescein (FITC) AffiniPure Goat Anti-Rabbit IgG (H+L)	111-095-003	Jackson ImmunoResearch, West Grove, PA, USA
Rhodamine (TRITC) AffiniPure Goat Anti-Rabbit IgG (H+L)	111-025-003	
Mouse Anti-β actin mAb	TA-09	ZSGB-Bio, Beijing, China

Appendix Table S2. Reagents were listed in this study

Reagents	No. of product	Company
Lipopolysaccharides from E.coli O111:B4 strain	L2630	Sigma, St. Louis, MO, USA
Ketamine	K2753	
Xylazine	X1126	
Polymyxin B sulphate	P1004	
Thioglycollate medium	T9032	
Bafilomycin A1	B1793	
MG132	C2211	
Phalloidin	P2495	
Hoechst33342	H1399	Invitrogen, Carlsbad, CA, USA
TRIzol	15596018	
DMEM	11995500	
LysoTracker™ Red DND-99	L7528	
Endothelial Cell Medium	1001	ScienCell Research Laboratories, Carlsbad, CA, USA
MTS	G1111	Promega, Madison, Wisconsin, USA
BSA	A8020	Solarbio Life Sciences, Beijing, China
BCA protein assay kit	PC0020	
Ficoll	P8900	
Eosin staining solution	G1100	
Trypticase Soy Agar	T8650	
rProtein A Sepharose Fast Flow	GE17-1279-03	GE Healthcare, Chicago, IL, USA
Protein G Sepharose 4 Fast Flow	GE17-0618-01	
Glutathione-Sepharose 4B	GE17-0756-01	
Proteinase inhibitor (PI) cocktail	5892970001	Roche Diagnostics GmbH, Penzberg, Germany
Phosphatase inhibitor (PPI) cocktail	4906845001	
In Situ Cell Death Detection Kit	11684795910	
Neomycin	0558	Amresco, Solon, OH, USA
Hematoxylin	ZLI-9610	ZSGB-Bio, Beijing, China

LEGENDplex™ mouse pro-inflammatory chemokine panel	740451	Biolegend Company, San Diego, CA, USA
RevertAid First Strand cDNA Synthesis Kit	K1622	Thermo Fisher Scientific, Waltham, MA, USA
SYBR Green qPCR Mix	Q131-02	Vazyme Biotech, Nanjing, Jiangsu, China
RIPA cell-lysis buffer<br>IP lysis buffer	P0013B<br>P0013	Beyotime Biotechnology, Shanghai, China
Immobilon Western HRP Substrate<br>Human plasma fibronectin	WBKLS<br>FC010	Merck, Darmstadt, Germany.
Neofect™ DNA transfection reagent	TF201201	Neofect biotech, Beijing, China
Recombinant Mouse ICAM-1<br>Recombinant Mouse M-CSF Protein<br>Recombinant Mouse IL-4 Protein	796-IC-050<br>416-ML-010<br>404-ML-010	R&D systems, Minneapolis, USA
Clodronate liposomes	CP-005	Liposoma BV, Amsterdam, The Netherlands

Appendix Table S3. Primers used for genome PCR and qRT-PCR

	Forward Primer	Reverse Primer
Genome PCR		
mTmem268	ATCGGAAGGTCAGCATTTA	TAGGCAGTGGCAGTCAAGC
qRT-PCR		
mTmem268	TGAGAGTGCCCTTTTGGAACC	GGCCCACACCAACACGTAG
mCD11b	ATGGACGCTGATGGCAATACC	TCCCCATTCACGTCTCCCA
mActb	GGCTGTATTCCCCTCCATCG	CCAGTTGGTAACAATGCCATGT
