# Supplementary material for: Deletion of Tmem268 in mice suppresses anti-infectious immune responses by downregulating CD11b signaling
Source: EMBO Rep. 2024 May 10;25(6):7. doi: 10.1038/s44319-024-00141-6 (PMC11169502; doi:10.1038/s44319-024-00141-6)
Supplement: Supplementary file 10 — Expanded View Figures [file 44319_2024_141_MOESM10_ESM.pdf]

Expanded View Figures

Figure EV1. *Tmem268* ablation exacerbates LPS- and CLP-induced inflammation, increases the bacterial burdens in reconstituted chimeric mice.

(**A**) Survival curve of *Tmem268*$^{+/+}$ and *Tmem268*$^{-/-}$ mice ($n = 5$) intraperitoneally injected with LPS (13 mg/kg). **P value = 0.0017, the Log-rank (Mantel–Cox) test. (**B**, **C**) Serum levels of TNF-α and IL-6 in mice intraperitoneally injected with LPS (5 mg/kg) for indicated time. Mean ± SD ($n = 3$). Unpaired two-tailed t test. For LPS 1 h, *P value = 0.0433. (**D**) H&E staining of *Tmem268*$^{+/+}$ and *Tmem268*$^{-/-}$ lung after LPS injection for 24 h. Scale bars = 50 μm. (**E**) Serum levels of TNF-α, IFN-β, MCP-1/CCL2 in mice after CLP surgery for 8 h. Mean ± SD ($n = 3$). Unpaired two-tailed t test. Mean ± SD. For TNF-α, ***P value = 0.0008. For IFN-β, ***P value = 0.0006. For MCP-1/CCL2, *P value = 0.0157. (**F**) The bacterial burdens were determined in different chimeric mice with CLP at 24 h. (**G**) H&E staining of lung in reconstituted chimeric mice with CLP at 24 h. Scale bars = 50 μm.

© The Author(s)

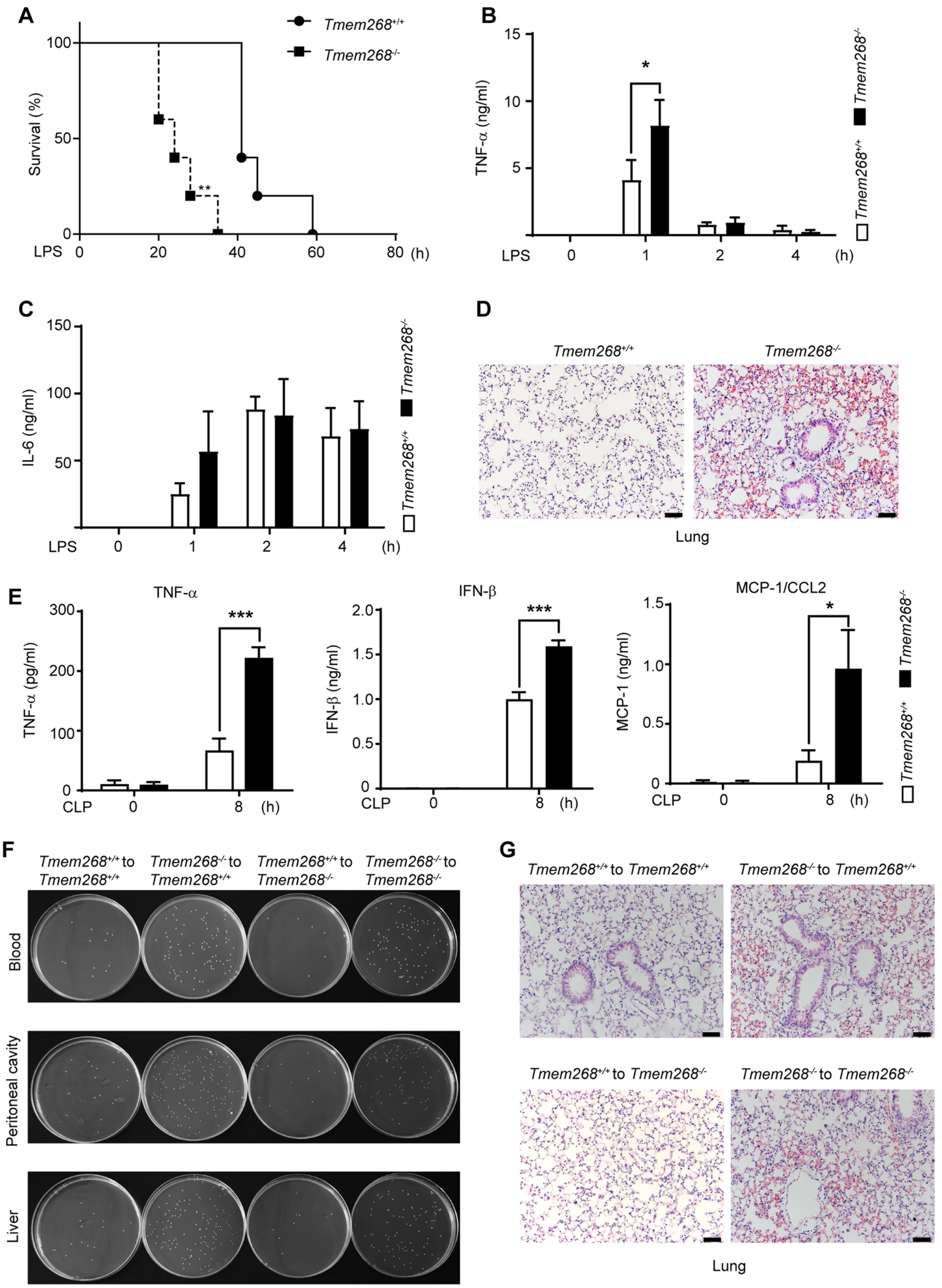
A
Tmem268+/+
Tmem268-/-
Survival (%)
LPS (h)
**
B
TNF-α (ng/ml)
*
C
IL-6 (ng/ml)
D
Lung
E
TNF-α
IFN-β
MCP-1/CCL2
TNF-α (pg/ml)
IFN-β (ng/ml)
MCP-1 (ng/ml)
CLP
***
F
Tmem268+/+ to Tmem268+/+
Tmem268-/- to Tmem268+/+
Tmem268+/+ to Tmem268-/-
Tmem268-/- to Tmem268-/-
Blood
Peritoneal cavity
Liver
G
Lung

 © The Author(s)

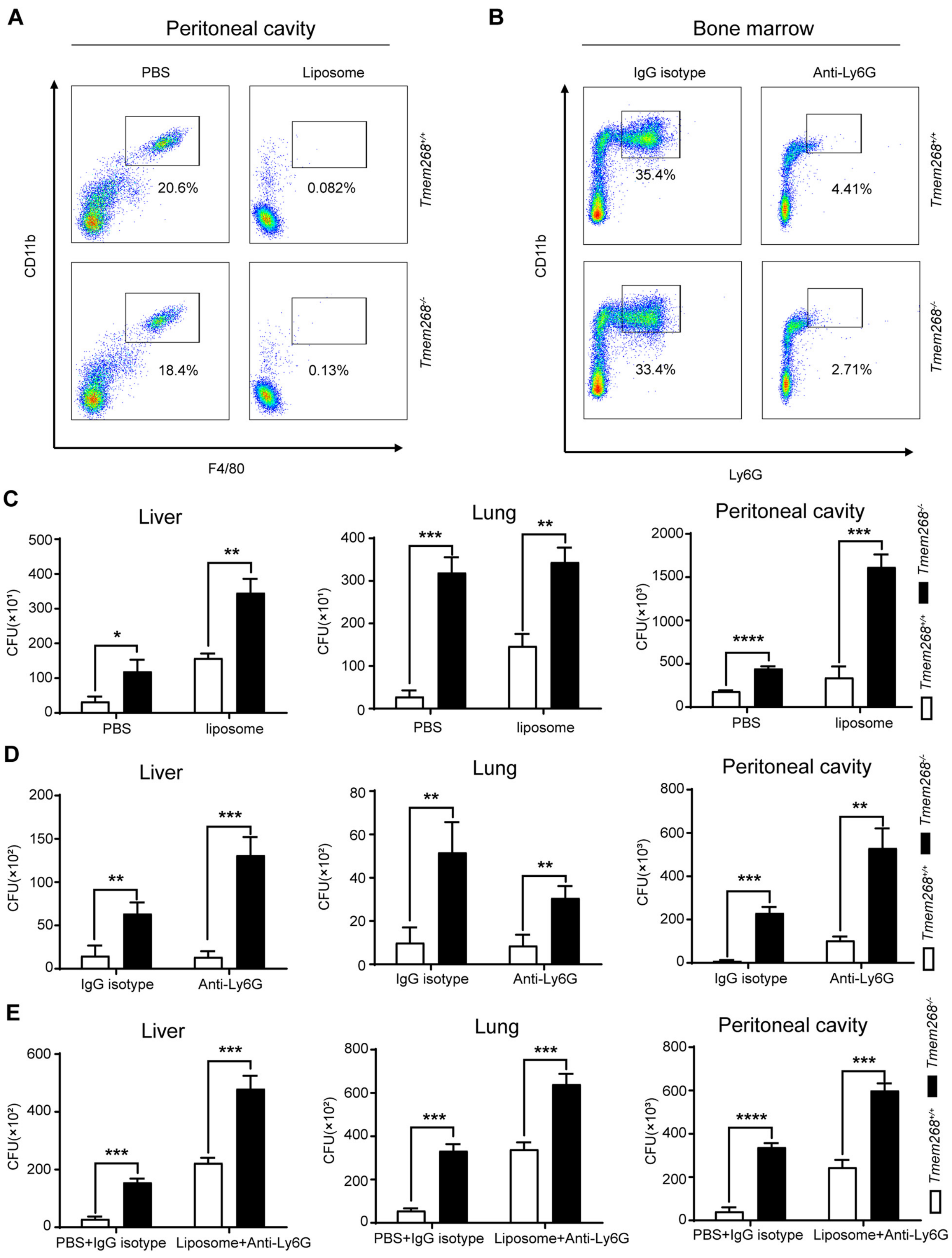
A
Peritoneal cavity
PBS
Liposome
20.6%
0.082%
18.4%
0.13%
CD11b
F4/80
Tmem268+/+
Tmem268-/-
B
Bone marrow
IgG isotype
Anti-Ly6G
35.4%
4.41%
33.4%
2.71%
CD11b
Ly6G
C
Liver
Lung
Peritoneal cavity
PBS
liposome
D
IgG isotype
Anti-Ly6G
E
PBS+IgG isotype
Liposome+Anti-Ly6G

Figure EV2. Both macrophages and neutrophils are involved in TMEM268-mediated antibacterial effects.

(**A**) The percentage of $CD11b^+F4/80^+$ macrophages in peritoneal cavity were analyzed by flow cytometry from *Tmem268*$^{+/+}$ and *Tmem268*$^{-/-}$ mice intraperitoneally injected with PBS or clodronate liposome at 72 h. (**B**) The percentage of $CD11b^+Ly6G^+$ neutrophils in bone marrow were analyzed by flow cytometry from *Tmem268*$^{+/+}$ and *Tmem268*$^{-/-}$ mice intraperitoneally injected with IgG isotype antibody or anti-Ly6G antibody at 48 h. (**C**) *Tmem268*$^{+/+}$ and *Tmem268*$^{-/-}$ mice pre-treated with PBS or clodronate liposome were subjected to CLP. 8 h later, the bacterial burdens in liver, lung and peritoneal cavity were measured. Mean ± SD ($n = 3$). Unpaired two-tailed t test. *$P < 0.05$, **$P < 0.01$, ***$P < 0.001$, ****$P < 0.0001$. (**D**) *Tmem268*$^{+/+}$ and *Tmem268*$^{-/-}$ mice pre-treated with IgG isotype or anti-Ly6G antibody were subjected to CLP. 8 h later, the bacterial burdens in liver, lung and peritoneal cavity were measured. Mean ± SD ($n = 3$). Unpaired two-tailed t test. **$P < 0.01$, ***$P < 0.001$, (**E**) *Tmem268*$^{+/+}$ and *Tmem268*$^{-/-}$ mice pre-treated with PBS+IgG isotype or liposome+anti-Ly6G antibody were subjected to CLP. 8 h later, the bacterial burdens in liver, lung and peritoneal cavity were measured. Mean ± SD ($n = 3$). Unpaired two-tailed t test. ***$P < 0.001$, ****$P < 0.0001$.

 © The Author(s)

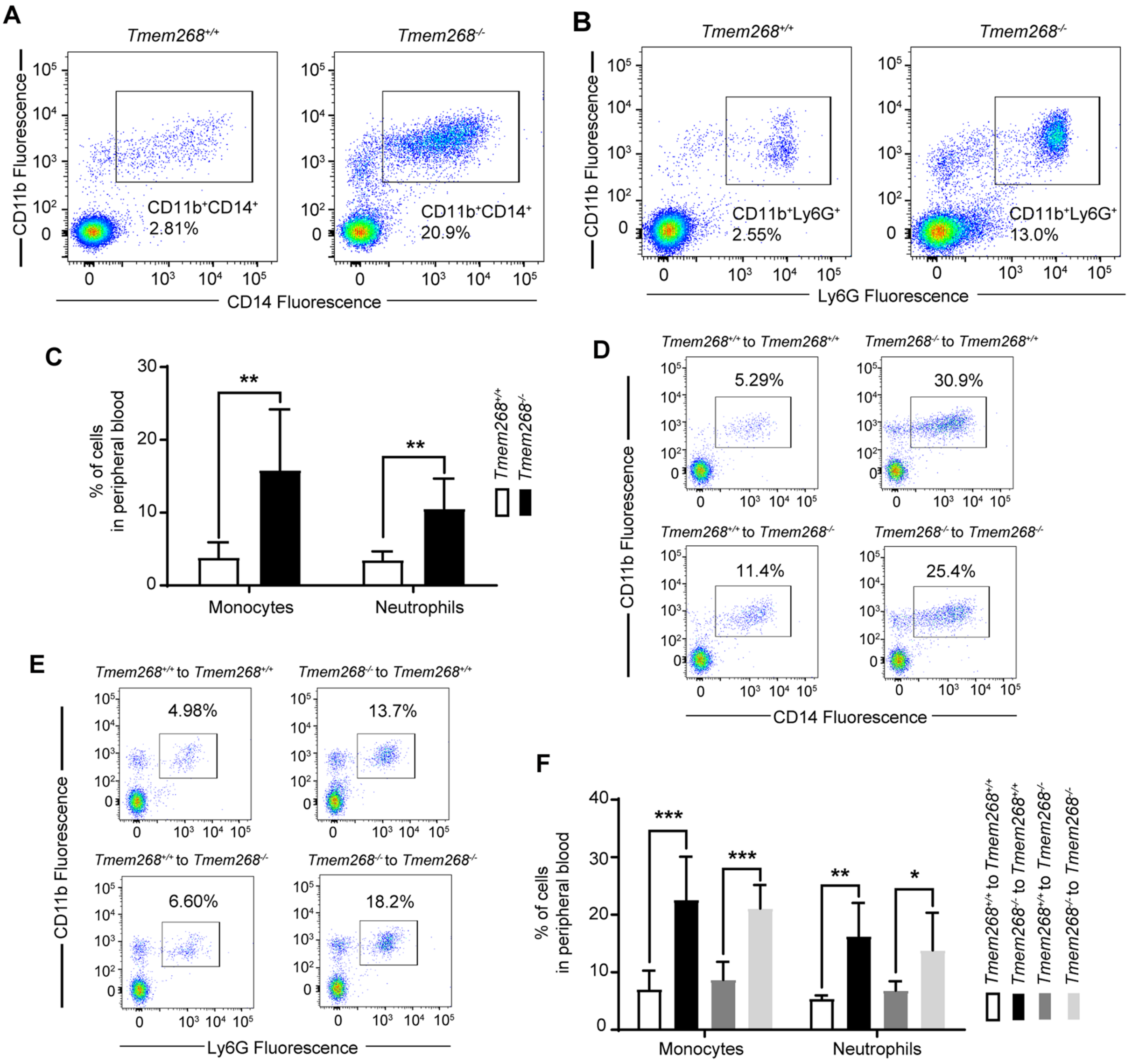

Figure EV3. ***Tmem268* deficiency increases the proportions of monocytes and neutrophils in peripheral blood.**

(**A**, **B**) The proportions of CD11b$^+$CD14$^+$ monocytes (**A**), CD11b$^+$Ly6G$^+$ neutrophils (**B**) in peripheral blood were detected by flow cytometry in *Tmem268*$^{+/+}$ and *Tmem268*$^{-/-}$ mice after CLP surgery for 8 h. (**C**) Quantification of monocytes and neutrophils in *Tmem268*$^{+/+}$ and *Tmem268*$^{-/-}$ mice after CLP surgery for 8 h. For monocytes, $^{**}P$ value = 0.0066. For neutrophils, $^{**}P$ value = 0.0026. Unpaired two-tailed *t* test. Mean ± SD ($n = 6$ mice). (**D**, **E**) The proportions of CD11b$^+$CD14$^+$ monocytes (**D**), CD11b$^+$Ly6G$^+$ neutrophils (**E**) in peripheral blood were detected by flow cytometry in *Tmem268*$^{+/+}$ and *Tmem268*$^{-/-}$ reconstituted chimeras after CLP surgery for 8 h. (**F**) Quantification of monocytes and neutrophils in *Tmem268*$^{+/+}$ and *Tmem268*$^{-/-}$ reconstituted chimeras after CLP surgery for 8 h. $^{*}P < 0.05$, $^{**}P < 0.01$, $^{***}P < 0.001$. Unpaired two-tailed *t* test. Mean ± SD ($n = 6$ mice).

© The Author(s)

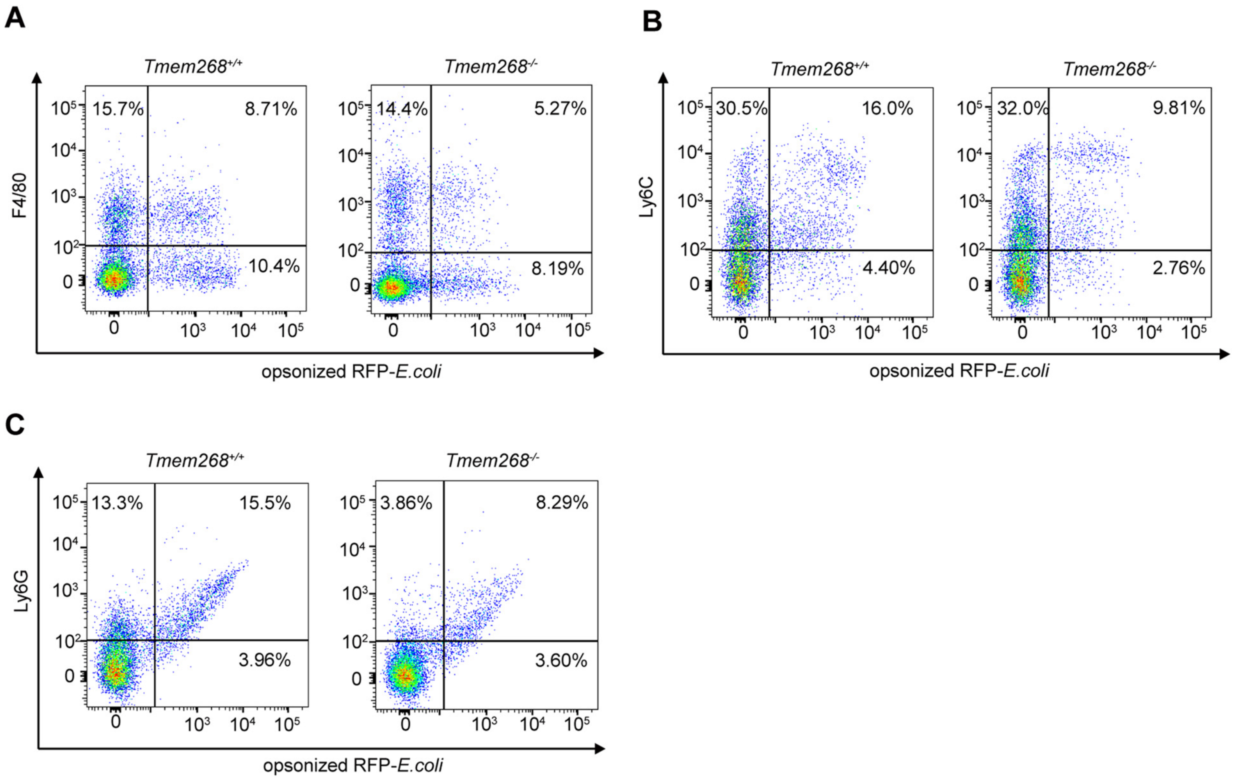

Figure EV4. *Tmem268* knockout impairs phagocytosis of serum-opsonized RFP-*E. coli*.

(**A–C**) *Tmem268*$^{+/+}$ and *Tmem268*$^{-/-}$ mice ($n = 5$) were intraperitoneally injected with LPS for 2 h, following injected with serum-opsonized RFP-*E.coli* for 30 min. Flow cytometry analysis of RFP$^+$F4/80$^+$(**A**), RFP$^+$Ly6C$^+$ (**B**), and RFP$^+$Ly6G$^+$ (**C**) cells in the abdominal cavity.

 © The Author(s)

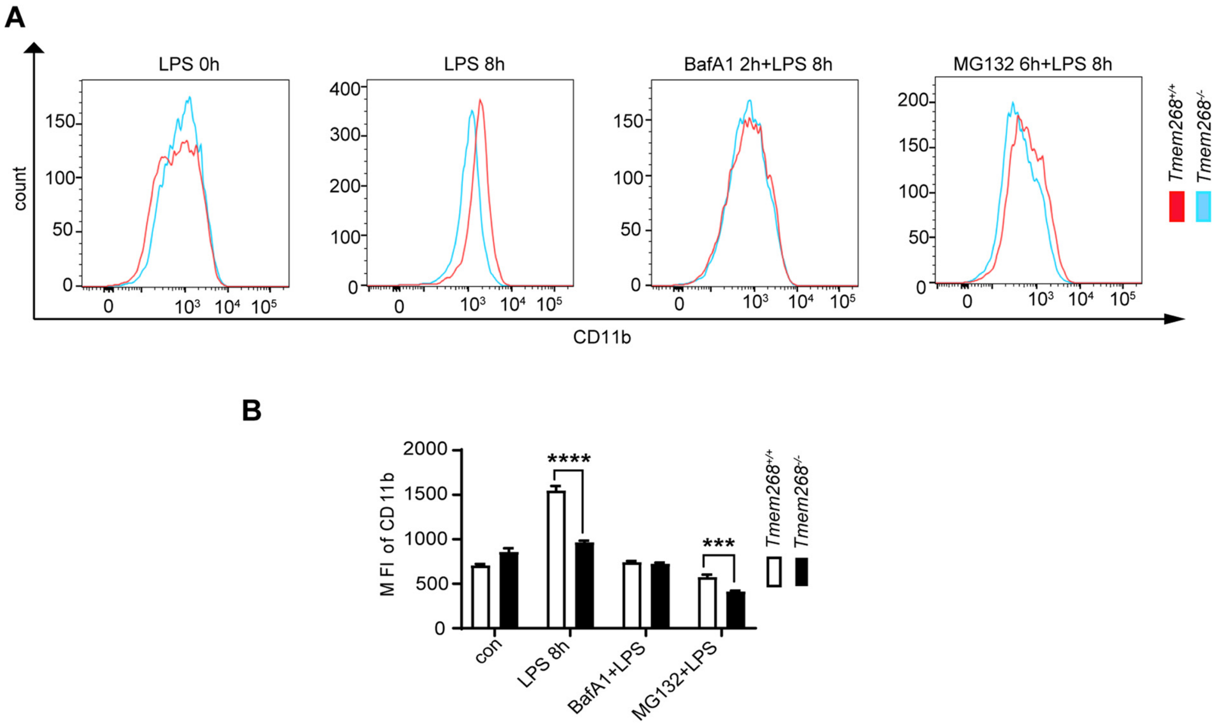

Figure EV5. *Tmem268* knockout promotes CD11b degradation via the lysosomal pathway.

(**A**) *Tmem268*$^{+/+}$ and *Tmem268*$^{-/-}$ BMDMs were treated as following: LPS (1 μg/ml) 8 h, or BafA1 (20 nmol/l) 2 h+ LPS (1 μg/ml) 8 h, or MG132 (10 μmol/l) 6 h+ LPS (1 μg/ml) 8 h. The membrane expression of CD11b was detected by flow cytometry. (**B**) The MFI of CD11b was statistically analyzed. For LPS 8 h, *****P* < 0.0001, For MG132 + LPS, ****P* value = 0.0007. Unpaired two-tailed *t* test. All plots represent mean ± SD from at least three independent experiments.
