# Supplementary material for: Deletion of Tmem268 in mice suppresses anti-infectious immune responses by downregulating CD11b signaling
Source: EMBO Rep. 2024 May 10;25(6):7. doi: 10.1038/s44319-024-00141-6 (PMC11169502; doi:10.1038/s44319-024-00141-6)
Supplement: Supplementary file 9 — Peer Review File [file 44319_2024_141_MOESM9_ESM.pdf]

EMBO reports

Deletion of Tmem268 in mice suppresses anti-infectious immune responses by downregulating CD11b signaling

Mengyuan Duan, Xuan Zhang1, Yaxin Lou, Jinqiu Feng, Pengli guo, Shufang Ye, Ping Lv, and Yingyu Chen

Corresponding author(s): Yingyu Chen (yingyu_chen@bjmu.edu.cn)

Review Timeline:

Submission Date:	24th May 23
Editorial Decision:	6th Jul 23
Revision Received:	2nd Jan 24
Editorial Decision:	29th Jan 24
Revision Received:	25th Feb 24
Accepted:	3rd Apr 24

Editor: Deniz Senyilmaz Tiebe

Transaction Report: No Peer Review Process File is available with this article, as the authors have chosen not to make the review process public.
